# Myeloid MyD88 restricts CD8^+^ T cell response to radiation therapy in pancreatic cancer

**DOI:** 10.1038/s41598-023-35834-w

**Published:** 2023-05-27

**Authors:** Terry R. Medler, Tiffany C. Blair, Alejandro F. Alice, Alexa K. Dowdell, Brian D. Piening, Marka R. Crittenden, Michael J. Gough

**Affiliations:** 1 Earle A. Chiles Research Institute, Providence Cancer Institute, Robert W. Franz Cancer Center, Providence Portland Medical Center, 4805 NE Glisan Street, Suite 2N100, Portland, OR 97213 USA; 2 The Oregon Clinic, Portland, OR USA

**Keywords:** Radiotherapy, Pancreatic cancer, Tumour immunology

## Abstract

Radiation therapy induces immunogenic cell death in cancer cells, whereby released endogenous adjuvants are sensed by immune cells to direct adaptive immune responses. TLRs expressed on several immune subtypes recognize innate adjuvants to direct downstream inflammatory responses in part via the adapter protein MyD88. We generated *Myd88* conditional knockout mice to interrogate its contribution to the immune response to radiation therapy in distinct immune populations in pancreatic cancer. Surprisingly, *Myd88* deletion in *Itgax* (CD11c)-expressing dendritic cells had little discernable effects on response to RT in pancreatic cancer and elicited normal T cell responses using a prime/boost vaccination strategy. *Myd88* deletion in *Lck*-expressing T cells resulted in similar or worsened responses to radiation therapy compared to wild-type mice and lacked antigen-specific CD8^+^ T cell responses from vaccination, similar to observations in *Myd88*^*−/−*^ mice. *Lyz2*-specific loss of *Myd88* in myeloid populations rendered tumors more susceptible to radiation therapy and elicited normal CD8^+^ T cell responses to vaccination. scRNAseq in *Lyz2-Cre/Myd88*^*fl/fl*^ mice revealed gene signatures in macrophages and monocytes indicative of enhanced type I and II interferon responses, and improved responses to RT were dependent on CD8^+^ T cells and IFNAR1. Together, these data implicate MyD88 signaling in myeloid cells as a critical source of immunosuppression that hinders adaptive immune tumor control following radiation therapy.

## Introduction

Successful immune responses require both antigen and adjuvant to provide immune targets and maturation signals that result in antigen presenting cell (APC) maturation, trafficking, cross presentation, and effective T cell costimulation. A range of pathogenic products can serve as immunological adjuvants, but these are mostly absent in the sterile immunity that occurs in response to tumor growth and treatment^1,2^. Instead, various modes of cell death can generate or release a range of endogenous immunological adjuvants that can provide signals to mature APCs and contribute to immune responses. While increased release of immunological adjuvants would be expected to enhance anti-tumor immunity, this doesn’t fit with the clinical data. Published data across a range of malignancies shows that necrosis is predictive of poor patient outcome^3–8^, that patients with increased expression of endogenous adjuvants such as calreticulin and HMGB1 in their tumor exhibit a worse prognosis than those with lower or absent expression^9,10^, and that blockade of the endogenous adjuvant HMGB1 improves immune control of tumors in preclinical models^11^. Despite this, cytotoxic therapies such as radiation therapy succeed where they increase endogenous adjuvant release^12–14^, and injection of a wide range of immune adjuvants at supraphysiological doses can improve immune control of tumors^1^. These data indicate that there is a disconnect between the potential positive role of immunological adjuvants, and their effects in tumorigenesis and conventional treatment. Importantly, the sensors for immunological adjuvants are not evenly spread among immune cells so that specific immune cells may specialize in sensing specific endogenous or exogenous adjuvants. In addition, the differentiation of immune cells can result in varying responses to the same adjuvant, as evidenced by the differently polarized macrophages associated with tumor progression and chronic immunity^15–17^.

TLRs are sensors that recognize microbial products and immunological adjuvants released by dying cells to direct downstream inflammatory reactions by MyD88-dependent and TRIF-dependent mechanisms [extensively reviewed in^18–20^]. All of the TLRs signal through the MyD88 pathway, with the exception of TLR3 which exclusively signals through TRIF, and TLR4 uniquely signals via both MyD88 and TRIF. Upon ligand engagement, TLRs dimerize to initiate signaling cascades downstream of these adaptor molecules, resulting in activation of NF-κB, AP-1, and IRF transcription factors that lead to activation of inflammatory programs. For the cell surface TLRs, the MyD88 pathway largely drives activation of NF-κB and AP-1, which leads to production of inflammatory cytokines, including TNFα, IL-1β, IL6, IL10, IL-12, and IL-18. Conversely, the TRIF/IRF pathway is largely responsible for production of type I IFN, but also converges on the NF-κB pathway to help control the balance of inflammatory gene expression and type I IFN production. Fine balance of these pathways downstream of TLR engagement in critical for pathogen clearance and avoidance of tissue pathology.

Seminal studies have revealed that TLRs expressed on antigen presenting cells, including Batf3^+^ dendritic cells (DCs), recognize endogenous adjuvants released by dying cells resulting in their maturation and enhanced cross-presentation to CD8^+^ T cells^21–30^. However, TLR signaling has also been shown to regulate macrophage phenotype and enhance T cell functionality by serving as a TCR costimulatory factor^19,31,32^. Because total *Myd88*^*−/−*^ mice lack critical immune functions of adjuvant signaling in each cell type described above, we generated conditional *Myd88* knockout mice to further understand how adjuvant sensing by TLRs within distinct immune subtypes regulates immune responses and tumor control following radiation therapy.

## Results

### *MYD88* and TLR expression in human and murine pancreatic cancer

**Figure 1:**
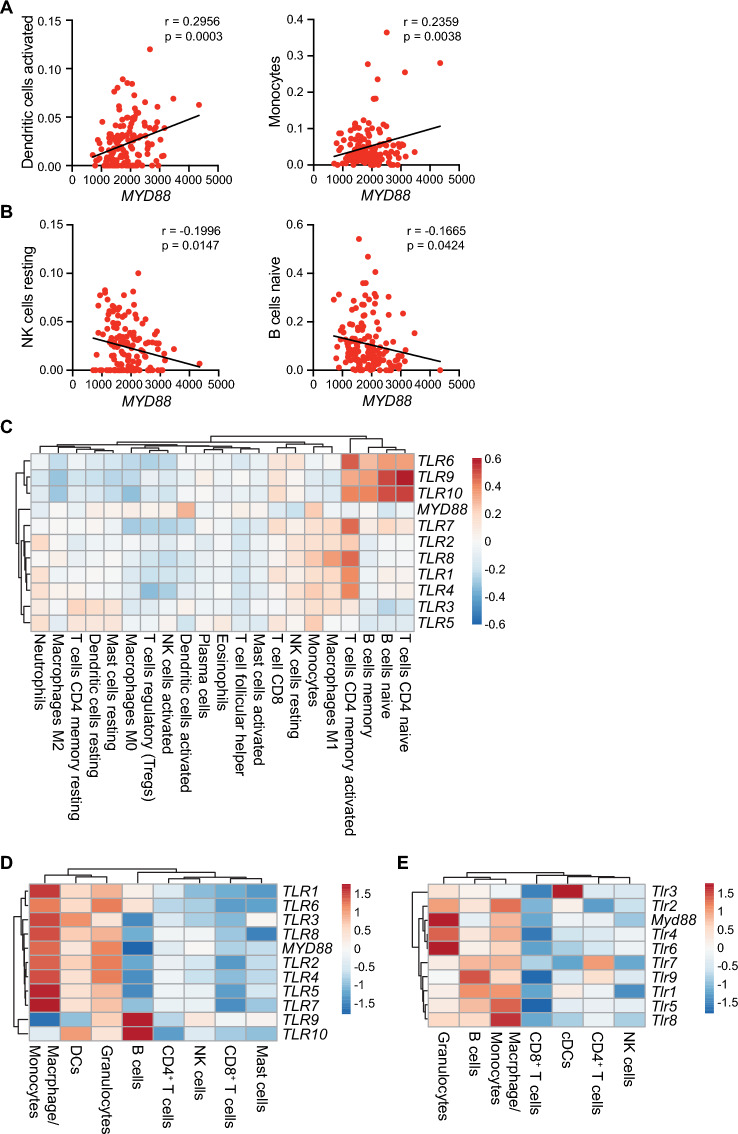
*MYD88* and *TLR* expression in human and murine pancreatic cancer. (**A**) Linear regression analysis showing positive (**A**) and negative (**B**) Pearson correlation between MYD88 expression and inferred presence of indicated immune subsets as estimated by CIBERSORT in the PAAD TCGA dataset (n = 149). (**C**) Heatmap showing linear regression analysis of TLR and MYD88 expression with presence of immune subsets as inferred by CIBERSORT in the PAAD TCGA dataset (n = 149). Positive Pearson correlations are indicated in shades of red, while negative correlations appear in blue. Data were visualized using ClustVis^35^. See Supplemental Fig. 1B for *p* values. (**D**) scRNAseq analysis on publicly available human PDAC samples^34^. Relative gene expression was assessed on *PTPRC*^+^ (CD45^+^) immune cells in PDAC, with non-immune cells excluded from analysis. Depicted are log_2_ fold change expression levels of indicated *TLR* genes and *MYD88* in *Itgam*^+^*Csf1r*^+^*CD68*^+^ macrophages/monocytes; *CD1A*^+^, *CD1B*^+^*, or CD1E*^+^ DCs; *CSF1R*^*-*^*CSF3R*^+^ granulocytes; *CD79A*^+^ B cells; *CD3E*^+^*CD4*^+^ CD4^+^ T cells; *CD3E*^*-*^*KLRC1*^+^ NK cells; *CD3E*^+^*CD8B*^+^ CD8^+^ T cells; and *CPA3*^+^ mast cells. (**E**) scRNAseq was performed on CD45^+^ cells isolated from untreated PK5L1940 tumors in *Myd88*^*fl/fl*^ mice (n = 4 mice). Depicted are log_2_ fold change expression levels of indicated *Tlr* genes and *Myd88* in *Itgam*^+^*Csf3r*^+^*Ly6c2*^*−*^ granulocytes, *Cd79b*^+^ B cells, *Itgam*^+^*Csf1r*^+^*Adgre*^+^ macrophages/monocytes, *Cd3e*^+^*Cd8a*^+^ CD8^+^ T cells, *Zbtb46*^+^ cDCs, *Cd3e*^+^*Cd4*^+^ CD4^+^ T cells, and *Klrb1c*^+^ NK cells. Significance in (**A**–**C**) was assessed by Pearson correlation, with *p* values as indicated in (**A**,**B**). See also Supplemental Fig. 1B.

Because multiple cell types within tumors utilize TLRs and MyD88 to respond to adjuvants released by cancer cells, we first sought to identify which cell types were associated with *MYD88* expression in the TCGA PAAD cohort. We utilized CIBERSORTx^33^ to correlate the presence of major immune subsets with *MYD88* expression. We found that *MYD88* expression was positively correlated with activated dendritic cells and monocytes (Fig. 1A), while its expression was negatively correlated with resting NK cells and naïve B cells (Fig. 1B). We found positive but insignificant associations with M0 and M2 macrophages, activated NK cells, mast cells, and Tregs, and negative, but insignificant association with M1 macrophages, memory resting CD4^+^ T cells, neutrophils, and CD8^+^ T cells (Supplemental Fig. 1A). We similarly used CIBERSORTx to determine correlation patterns between TLR expression and presence of cell types. We found that B cells, naïve and memory activated CD4^+^ T cells formed a cluster of high positive correlation with *TLR6*, *TLR9*, and *TLR10* expression and slight negative correlation with *TLR3* and *TLR5* expression (Fig. 1C and Supplemental Fig. 1B). Forming a cluster of positive correlation amongst expression of most TLRs were M1 macrophages, monocytes, and to a lesser extent, resting NK cells and CD8 T cells (Fig. 1C and Supplemental Fig. 1B). There was a slight negative correlation between TLR expression and presence of activated mast cells, Tfh cells, eosinophils, and plasma cells, with a more robust negative correlation between TLR expression and presence of activated DCs and NK cells, Tregs, and M0 macrophages (Fig. 1C and Supplemental Fig. 1B). Forming a cluster of more heterogeneous TLR expression were resting mast cells, resting DCs, memory resting CD4^+^ T cells, M2 macrophages, and neutrophils (Fig. 1C and Supplemental Fig. 1B). To further verify *TLR* and *MYD88* expression in human PDAC, we assessed their expression in a publicly available scRNAseq dataset^34^. We filtered our analysis to *PTPRC*^+^ (CD45^+^) immune cells in PDAC tissue and excluded all other cell types from the analysis. We found that *MYD88* was highly expressed in macrophages/monocytes, DCs, and granulocytes (Fig. 1D). Macrophages/monocytes expressed very high levels of all *TLR*s, apart from *TLR9* to*TLR10* (Fig. 1D). Granulocytes expressed high levels of all *TLR*s, while DCs expressed similarly high levels of all *TLR*s except for *TLR9* (Fig. 1D). B cells expressed very high levels of *TLR9* and *TLR10* and lower levels of *TLR1* and *TLR6*, while NK cells expressed low levels of *TLR9* (Fig. 1D). All other immune cells expressed relatively low levels of *MYD88* and *TLRs*.

We next determined which cell types express *Myd88* and TLRs in murine pancreatic tumors. To do this, we performed single-cell RNA sequencing (scRNAseq) on CD45^+^ cells isolated from untreated PK5L1940 tumors in wild-type mice. We found that macrophages/monocytes, B cells, and granulocytes expressed the highest levels of TLRs overall (Fig. 1E). Granulocytes expressed highest levels of *Tlr6*, *Tlr4*, and *Tlr2*, while macrophages/monocytes expressed highest levels of *Tlr8*, *Tlr5*, and *Tlr2* (Fig. 1E). Conversely, T cells, NK cells, and cDCs expressed lower levels of most TLRs, except cDCs expressed very high levels of *Tlr3*, which does not signal through MyD88 (Fig. 1E). Notably, granulocytes and macrophages/monocytes expressed the highest levels of *Myd88* (Fig. 1E). Together, these data indicate that *MYD88* and TLRs are heterogeneously expressed in pancreatic cancer, with granulocytes, B cells, and cells of the macrophage/monocyte lineage expressing high levels of TLRs that signal through MyD88.

### Myeloid MyD88 restricts antitumor response to RT in pancreatic cancer

**Figure 2:**
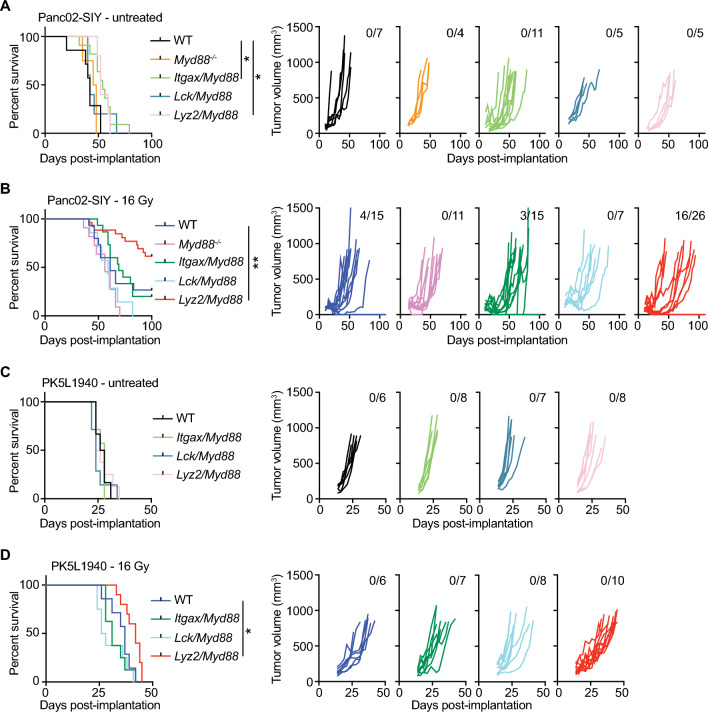
Myeloid MyD88 restricts response to RT in pancreatic cancer. (**A**) Survival curves (left) and individual Panc02-SIY tumor growth curves (right) in untreated mice of the indicated genotype (n = 4–11 mice per group). (**B**) Survival curves (left) and individual Panc02-SIY tumor growth curves (right) in mice of the indicated genotype treated with 16 Gy RT (n = 7–26 mice per group). (**C**) Survival curves (left) and individual PK5L1940 tumor growth curves (right) in untreated mice of the indicated genotype (n = 6–8 mice per group). (**D**) Survival curves (left) and individual PK5L1940 tumor growth curves (right) in mice of the indicated genotype treated with 16 Gy RT (n = 7–10 mice per group). Numbers in the upper right of individual tumor growth curves in (**A**–**D**) represent the proportion of mice cured by RT. Significance in (**A**–**D**) was assessed by log-rank test. * *p* < 0.05; ** *p* < 0.01.

*Myd88* knockout in all host cells has been associated with defective T cell responses correlated with impaired DC maturation and cross presentation^21,33^. However, our scRNAseq results indicated that macrophages/monocytes and granulocytes may be primed to respond to TLR signals, given that they express high levels of *Myd88* and several *Tlr*s. In order to delineate the effects of MyD88 within distinct immune subsets, we crossed *Myd88*^*fl/fl*^ mice with *Itgax*(CD11c)*-Cre* mice (primarily DC-specific), *Lck-Cre* mice (T cell-specific), or *Lyz2-Cre* mice (granulocyte-, macrophage-, and monocyte-specific). To determine how cell type-specific loss of *Myd88* expression affects tumor growth and responses to radiation therapy (RT), we used two pancreatic cell lines Panc02-SIY and the more aggressive PK5L1940. Each have the SIY model antigen, but while Panc02 arose following MCA mutagenesis of the murine pancreas^36^, PK5L1940 developed from a KPC-LSIY genetically engineered murine model^37^. 14 days post-implantation of Panc02-SIY cells, mice were left untreated or were treated with 16 Gy RT and overall survival was assessed. In untreated mice, we found moderately improved outcomes in both *Itgax-Cre/Myd88*^*fl/fl*^ and *Lyz2-Cre/Myd88*^*fl/fl*^ mice, implicating MyD88 as an immunosuppressive factor in both DCs and myeloid cells during tumor growth (Fig. 2A). In response to RT, *Myd88*^*−/−*^ mice had comparable outcomes compared to *Myd88*^*fl/fl*^ mice, paralleling prior reports^21,38^ (Fig. 2B). Similarly, *Itgax-Cre/Myd88*^*fl/fl*^ mice had outcomes that were not significantly different compared to control *Myd88*^*fl/fl*^ mice treated with RT (Fig. 2B). *Lck-Cre/Myd88*^*fl/fl*^ mice consistently had slightly larger tumors at time of treatment, though this did not impact survival as outcomes were similar to *Myd88*^*fl/fl*^ mice (Fig. 2A,B). However, *Lyz2-Cre/Myd88*^*fl/fl*^ mice had significantly improved outcomes in response to RT, with tumor cures observed in 61.5% of mice (Fig. 2B). Flow cytometric analysis of tumors in WT and *Lyz2-Cre/ Myd88*^*fl/fl*^ mice 7d post-RT revealed that broad populations of infiltrating immune cells were largely unchanged between groups, with the exception that CD11b^+^MHCII^*−*^Ly6C^*−*^Ly6G^−^ immature myeloid cells were increased in *Lyz2-Cre/ Myd88*^*fl/fl*^ mice treated with RT (Supplemental Fig. S2). To validate these data in another model, we implanted the more aggressive pancreatic cancer cell line PK5L1940 into mice. We found no differences among any genotype in untreated mice, but again found improved survival in *Lyz2-Cre/Myd88*^*fl/fl*^ mice post-RT (Fig. 2C,D). Together, these data indicate that loss of MyD88 in the myeloid compartment improves pancreatic cancer response to RT, which cannot be observed with total MyD88 loss.

### Improved survival in ***Lyz2-Cre/Myd88***^***fl/fl***^ mice correlates with de-repression of interferon responses

**Figure 3:**
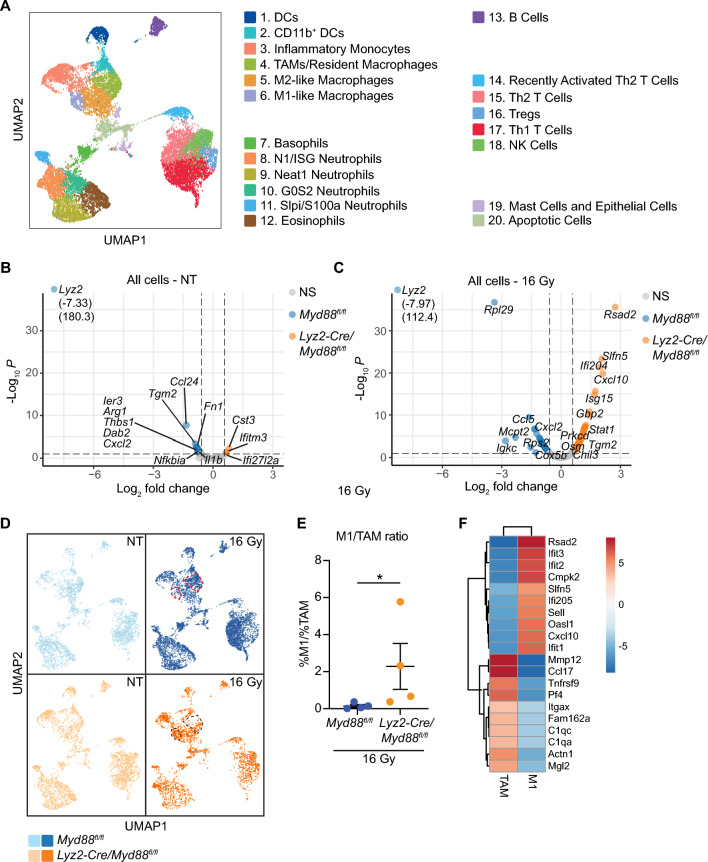
scRNAseq in untreated and RT-treated tumors from *Myd88*^*fl/fl*^ and *Lyz2-Cre/Myd88*^*fl/fl*^ mice. scRNAseq was performed on CD45^+^ cells isolated from untreated tumors or 3d post-RT in *Lyz2-Cre/Myd88*^*fl/fl*^ mice and *Myd88*^*fl/fl*^ mice (n = 4 mice per group). (**A**) Cells were subjected to graph-based clustering using the Loupe Cell Browser, with the UMAP plot of unsupervised clustering shown. Dominant cell types within each cluster were identified using expression of known markers (see also Supplemental Fig. S3,S4). (**B**,**C**) Volcano plots representing a global view of differential gene expression in CD45^+^ cells that are upregulated in *Lyz2-Cre/Myd88*^*fl/fl*^ mice (right, orange) or upregulated in *Myd88*^*fl/fl*^ mice (left, blue) in untreated mice (**B**) or mice treated with 16 Gy RT (**C**). (**D**) UMAPs were split according to genotype and treatment, with cells from *Lyz2-Cre/Myd88*^*fl/fl*^ mice identified in orange and *Myd88*^*fl/fl*^ mice identified in blue. Untreated mice are depicted in the lighter shade, while mice treated with 16 Gy RT are shown in the darker shade. TAM and M1 macrophage clusters are circled in either red or black in 16 Gy treated mice. (**E**) Graph depicting the ratio of M1:TAM populations as a percentage of total immune cells in *Lyz2-Cre/Myd88*^*fl/fl*^ mice treated with 16 Gy compared to *Myd88*^*fl/fl*^ mice treated with 16 Gy. (**F**) Top 10 genes upregulated in M1 and TAM populations. Significance of differential gene expression in (**B**,**C**) was defined as fold change > 1.5 and *p* < 0.1. Significance in (**E**) was determined by Mann–Whitney test, * *p* < 0.05.

To understand the improved response to RT in *Lyz2-Cre/Myd88*^*fl/fl*^ mice compared to *Myd88*^*fl/fl*^ mice and to assess the tumor immune phenotypes in the in vivo setting, we performed single cell RNA sequencing on CD45^+^ immune cells isolated from PK5L1940 tumors in untreated mice and 3d post-RT. UMAPs were generated in an unbiased manner and revealed that immune populations largely grouped according to dominant cell types present within each cluster according to lineage marker expression, with subpopulations of DCs, macrophages, and monocytes representing one large cluster, subpopulations of granulocytes representing a second large cluster, and several populations of T and NK cells representing a third large cluster (Fig. 3A). Among these clusters, we observed two different populations of DCs, four populations of macrophages/monocytes, four populations of neutrophils, and four populations of T cells (Fig. 3A and Supplemental Fig. S3,4). Macrophages and monocytes expressed of high levels of *Itgam* (CD11b), *Csf1r*, *Ccr2*, and *Adgre* (F4/80). Within these populations, monocytes were in part defined by expression of high levels of *Cxc3r1*; M1 macrophages had increased expression of interferon responsive genes, including *Isg15*, *Cxcl10*, *Ifit1*, *Ifit2*, and *Ifit3*; M2-like macrophages expressed high levels of *Vegfa*, *Arg1*, and *Mrc1*; while TAMs/Tissue resident macrophages also expressed these genes at high levels but also expressed high levels of C1q transcripts (Supplemental Fig. S2,3). Differential gene expression analysis amongst the entire immune population of untreated mice revealed decreased levels of factors associated with immunosuppression and recruitment of immunosuppressive cells, including *Arg1*, *Tgm2*, *Ccl24*, and *Cxcl2* in *Lyz2-Cre/Myd88*^*fl/fl*^ mice compared to control mice (Fig. 3B). In RT-treated mice, we saw increased expression of several genes associated with type I and type II interferon responses, including *Cxcl10*, *Stat1*, *Isg15*, and *Ifi204* and decreased expression of factors associated with chemotaxis, including *Cxcl2 and Ccl5* in *Lyz2-Cre/Myd88*^*fl/fl*^ mice compared to control mice (Fig. 3C). Separation of UMAPs according to genotypes and treatment groups revealed more striking differential gene expression between *Myd88*^*fl/fl*^ and *Lyz2-Cre/Myd88*^*fl/fl*^ in mice treated with RT compared to untreated mice (Fig. 3D). Aside from the lack of both Th1 and Th2 T cells in *Lyz2-Cre/Myd88*^*fl/fl*^ mice 3d post-RT, which may be due to reduced levels of *Ccl5* at this timepoint, we observed differences in the types of macrophages present within tumors. There was a notable increase in the M1/TAM ratio in *Lyz2-Cre/Myd88*^*fl/fl*^ mice compared to *Myd88*^*fl/fl*^ mice (Fig. 3D,E). Differential gene expression of the top 10 genes between the M1 and TAM populations revealed an increase in interferon responsive genes in the M1 population and increased factors associated with immunosuppression and opsonization in the TAM population (Fig. 3F).

**Figure 4:**
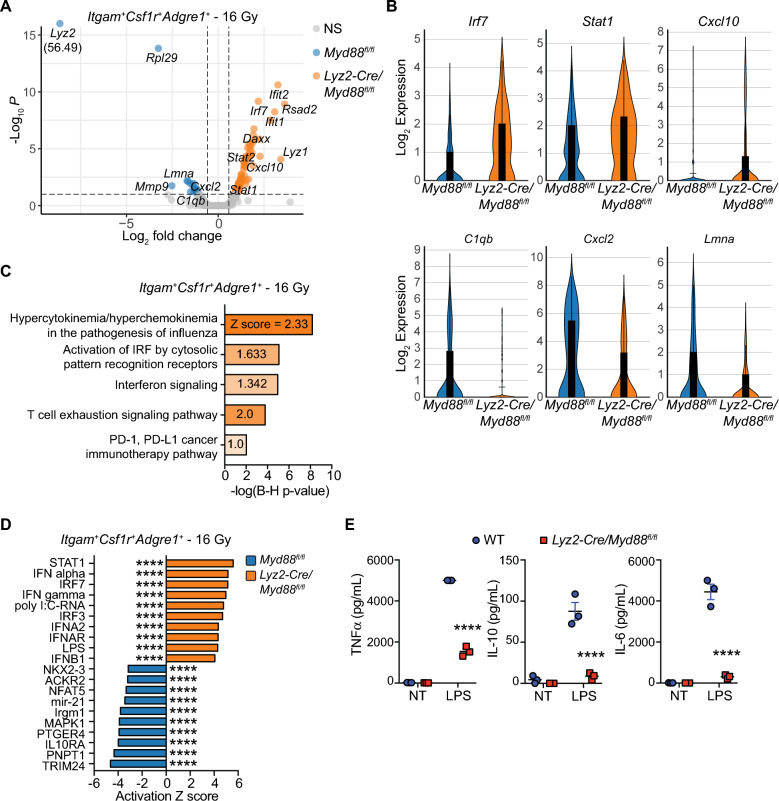
Enhanced interferon responses characterize infiltrating macrophages and monocytes in tumors from *Lyz2-Cre/Myd88*^*fl/fl*^ mice. scRNAseq was performed on CD45^+^ cells isolated from untreated tumors or 3d post-RT in *Lyz2-Cre/Myd88*^*fl/fl*^ mice and *Myd88*^*fl/fl*^ mice (n = 4 mice per group). (**A**) Volcano plot of differential gene expression in macrophages and monocytes (*Itgam*^+^*Csf1r*^+^*Adgre1*^+^ cells) that are upregulated in *Lyz2-Cre/Myd88*^*fl/fl*^ mice (right, orange) or upregulated in *Myd88*^*fl/fl*^ mice (left, blue). (**B**) Violin plots of select genes from (**A**). (**C**) Canonical pathway analysis using Ingenuity Pathway Analysis extrapolating likely pathways activated in *Lyz2-Cre/Myd88*^*fl/fl*^ mice treated with 16 Gy RT based off of significant differential gene expression from (**A**). (**D**) Upstream analysis based off of significant differential gene expression from (**A**) using Ingenuity Pathway Analysis, which identifies likely regulators that explain differential gene expression patterns observed in *Lyz2-Cre/Myd88*^*fl/fl*^ mice (top, orange) or *Myd88*^*fl/fl*^ mice (bottom, blue). (**E**) Quantitation of TNFα (left), IL-10 (center), and IL-6 (right) in supernatants of BMMΦs of the indicated genotype 24 h after treatment with 100 ng/mL LPS as measured by the ProcartaPlex platform. Significance of differential gene expression in (**A**) was defined as fold change > 1.5 and *p* < 0.1. Significance in (**E**) was assessed by an unpaired, two-sided *t*-test. * *p* < 0.05, ** *p* < 0.01, **** *p* < 0.0001.

To further understand which cell types and inflammatory programs are driving improved responses to RT in *Lyz2-Cre/Myd88*^*fl/fl*^ mice, we assessed differential gene expression patterns in distinct immune populations within RT-treated mice. *Lyz2* expression is highest among macrophages and monocytes, with reduced expression amongst granulocytes (Supplemental Fig. S5A). Differential gene expression analysis of significantly regulated genes in *Myd88*-deficient *Itgam*^+^*Csf1r*^+^*Adgre1*^+^ (CD11b^+^CSF1R^+^F4/80^+^) cells (all macrophages/monocytes) revealed increased gene expression of interferon-responsive genes (e.g. *Ifit1/2*, *Irf7, Stat1/2,* and *Cxcl10),* and decreased expression of some genes associated with Th2/M2-type macrophages (e.g. *Mmp9*, *Lmna*, *Stat1*) and recruitment of tumor-associated neutrophils (*Cxcl2)*^39^ (Fig. 4A,B). Network analysis using Ingenuity Pathway Analysis (IPA) revealed a gene expression network indicative of increased type I IFN, including genes associated with increased MHC class I loading, as well as genes directly or indirectly associated with IRF7 and STAT1/2 signaling (Supplemental Fig. S5B). IPA canonical pathway analysis revealed gene signatures associated with cytokine and chemokine expression in influenza, activation of IRF by cytosolic PRRs, interferon signaling, and genes associated with T cell exhaustion/function (Fig. 4C). Upstream analysis indicated increased gene expression patterns associated with activation of STAT1, IRF3 and IRF7, type I and II interferons, and decreased expression patterns associated with Th2/M2-type macrophages including TRIM24, IL10RA, and NFAT5 (Fig. 4D). *Itgam*^+^*Csf3r*^+^*Ly6c2-*granulocytes (Supplemental Fig. S6A,B) and *Zbtb46*^+^ cDCs (Supplemental Fig. S6C,D) were affected in tumors to a lesser extent than macrophages and monocytes, but similarly expressed gene expression patterns associated with type I and type II interferons and genes associated with PRR signaling. Given our scRNAseq data above indicating that macrophage phenotype was changed in *Lyz2-Cre/Myd88*^*fl/fl*^ mice responding to RT, we determined how TLR4 ligation with LPS affected cytokine output from bone marrow-derived macrophages (BMMΦs) from *Myd88*^*fl/fl*^ or *Lyz2-Cre/Myd88*^*fl/fl*^ mice to mimic adjuvant release in a more controlled setting. We found that BMMΦs from *Lyz2-Cre/Myd88*^*fl/fl*^ mice stimulated with LPS had significantly reduced TNFα, IL-10, and IL-6 production compared to control BMMΦs (Fig. 4E). Altogether, these data indicate that *Myd88* expression in myeloid cells, including macrophages, results in a suppressive phenotype in macrophages responding to TLR ligation and repression of IFN production.

### T cell MyD88 is required for antigen-specific responses

**Figure 5:**
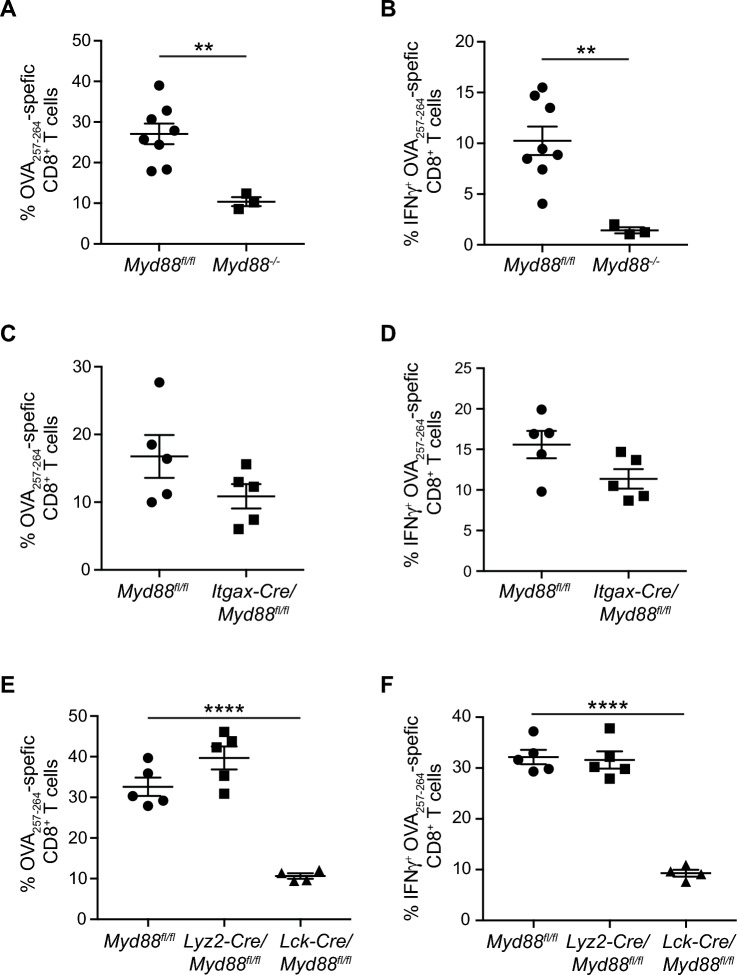
*Myd88* expression in T cells is required for antigen-specific CD8^+^ T cell responses to vaccination. (**A**,**C**,**E**) Flow cytometric analysis of Ova-specific CD8^+^ T cells in splenocytes isolated from *Myd88*^*−/−*^ mice (**A**), *Itgax-Cre/Myd88*^*fl/fl*^ mice (**C**), or *Lck-Cre/Myd88*^*fl/fl*^ mice and *Lyz2-Cre/Myd88*^*fl/fl*^ mice (**E**) compared to *Myd88*^*fl/fl*^ mice after vaccination with *∆ActA-Ova L. monocytogenes*. (**B**,**D**,**F**) Same as in (**A**,**C**,**E**, respectively), except splenocytes were stimulated with Ova_257–264_ (SL8) peptide prior to flow cytometric analysis in the presence of brefeldin A to assess IFNγ positivity in Ova-specific CD8^+^ T cells. Each data point represents a single mouse (n = 3–8 mice per group). Significance was assessed by an unpaired, two-sided *t*-test (**A**–**D**), and one-way ANOVA (**E**,**F**). Data are represented as means ± SEM. ** *p* < 0.01; **** *p* < 0.0001.

Type I IFN production is important for T cell responses^40,41^ and the results above indicate that RT fails to induce type I IFN when myeloid cells express MyD88. However, T cell expression of MyD88 has also been shown to be important for CD8^+^ T cell responses^42–44^. We therefore sought to better understand how T cell function is affected when MyD88 is deleted within distinct immune subsets. To assess this, we utilized a prime/boost vaccination strategy with *∆ActA*-Ova *Listeria monocytogenes*. In control mice, we observed robust CD8^+^ T cell responses with ample interferon gamma (IFNγ) production (Fig. 5A,B). This response was lost in total *Myd88*^*−/−*^ mice where mice displayed an inability to control infection, with 7/10 mice succumbing to infection (Fig. 5A,B). In *Itgax-Cre/Myd88*^*fl/fl*^ mice, we observed a modest but insignificant decrease in antigen specific CD8^+^ T cell responses and IFNγ production, indicating that DC expression of MyD88 is not essential for antigen-specific CD8^+^ T cell responses in this context (Fig. 5C,D). However, T cell-specific loss of MyD88 (*Lck-Cre/Myd88*^*fl/fl*^) largely recapitulated the results observed in total *Myd88*^*−/−*^ mice, with deficient T cell responses to vaccination (Fig. 5E,F). Importantly, myeloid-specific *Myd88* deletion (*Lyz2-Cre/Myd88*^*fl/fl*^) had no significant effects on antigen specific CD8^+^ T cell responses, which were similar to *Myd88*^*fl/fl*^ mice (Fig. 5E,F). These data are consistent with reports demonstrating that loss of MyD88 in T cells restrains their function. For example, adoptive transfer experiments using *Myd88*^*−/−*^ CD8^+^ T cells demonstrated their inability to undergo clonal expansion in response to LCMV infection, though their effector functions were unchanged^45^. The retained function in CD8^+^ T cells in this case may be due to intact MyD88 signaling in CD4^+^ T cells. Indeed, MyD88 signaling in CD4^+^ T cells is also important for CD8^+^ T cell function, as demonstrated by a lack of primary expansion and loss of effector function in mice lacking both CD4^+^ and CD8^+^ T cells with or without antigen-specific stimulation^46,47^, and T cell responses to a range of infectious models^45,48–53^. Together, these results indicate that *Myd88* expression in T cells is critical for antigen-specific CD8^+^ T cell responses and effector responses, which likely influenced results from tumor studies performed in total *Myd88*^*−/−*^ mice.

### Improved survival in *Lyz2-Cre/Myd88*^*fl/fl*^ mice is dependent on CD8^+^ T cells and Type I IFN

**Figure 6:**
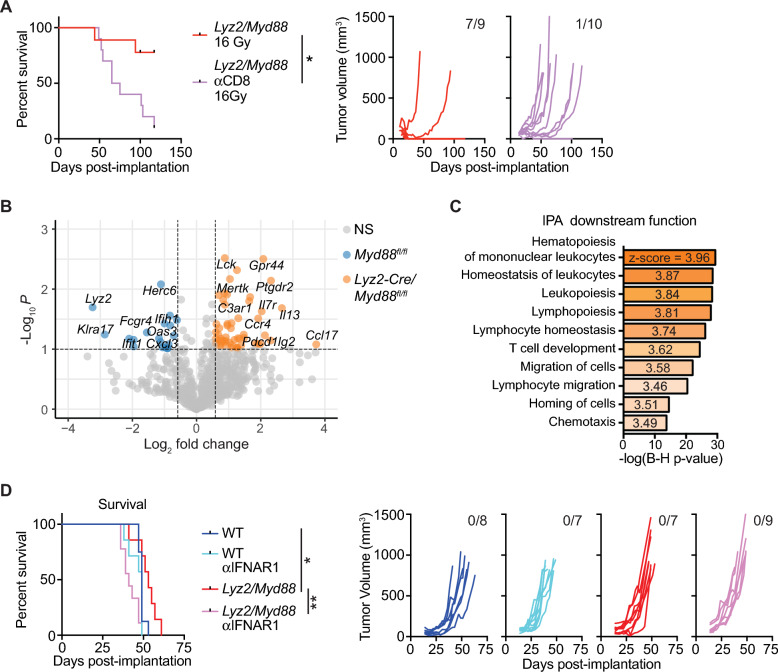
Improved response to RT in *Lyz2-Cre/Myd88*^*fl/fl*^ mice is dependent on CD8^+^ T cells and Type I IFN. (**A**) Survival curves (left) and individual Panc02-SIY tumor growth curves (right) in *Lyz2-Cre/Myd88*^*fl/fl*^ mice treated with 16 Gy RT and αCD8 mAb (n = 9–10 mice per group). Numbers in the upper right of individual tumor growth curves represent the proportion of mice cured by RT. (**B**,**C**) Differential gene expression analysis of Panc02-SIY whole tumor lysates from *Lyz2-Cre/Myd88*^*fl/fl*^ mice and *Myd88*^*fl/fl*^ mice treated with 16 Gy RT as determined by NanoString analysis using the PanCancer Immune Profiling gene set (n = 2 mice per group; experiment performed once). (**B**) Volcano plot of genes significantly upregulated (fold change > 1.5, *p* < 0.1) in *Lyz2-Cre/Myd88*^*fl/fl*^ mice (right, orange; n = 2) or upregulated in *Myd88*^*fl/fl*^ mice (left, blue; n = 2). (**C**) Ingenuity Pathway Analysis of downstream functions likely resulting from differences in gene expression in *Lyz2-Cre/Myd88*^*fl/fl*^ mice compared to *Myd88*^*fl/fl*^ mice. (**D**) Survival curves (top) and individual PK5L1940 tumor growth curves (bottom) in *Myd88*^*fl/fl*^ (WT) mice and *Lyz2-Cre/Myd88*^*fl/fl*^ mice treated with 16 Gy RT and αIFNAR1 mAb (n = 7–9 mice per group). Numbers in the upper right of individual tumor growth curves represent the proportion of mice cured by RT. Significance in (**A** and **D**) was determined by log-rank test. Significance of differential gene expression in (**B**,**C**) was defined as fold change > 1.5 and *p* < 0.1, with the experiment performed once. Data are represented as means ± SEM. * *p* < 0.05; **** *p* < 0.0001.

Given the normal T cell function in response to vaccination in *Lyz2-Cre/Myd88*^*fl/fl*^ and the results above indicating increased type I IFN production, we wanted to determine whether improved survival was dependent on CD8^+^ T cells. Despite being a poorly radioimmunogenic tumor model in wild-type mice, we found that depletion of CD8^+^ T cells reversed the efficacy of RT on Panc02-SIY tumors in *Lyz2-Cre/Myd88*^*fl/fl*^ mice (Fig. 6A). Thus, while CD8^+^ T cell depletion has no effect on response to RT in wild-type mice using this tumor model^54,55^, these data indicate that the improved response to RT with loss of MyD88 in myeloid cells is dependent on T cell function. To better understand the effects of RT in *Lyz2-Cre/Myd88*^*fl/fl*^ mice on the tumor environment following treatment, we performed NanoString analysis using the Pan-Cancer Immune Profiling panel of whole tumors 7d post-RT, which revealed increased gene expression signatures indicative of leukopoiesis/lymphopoiesis, lymphocyte chemotaxis, and T cell development when myeloid cells lack MyD88 (Fig. 6B,C and Supplemental Fig. S7A).

Given that multiple cell types lack *Myd88* in *Lyz2-Cre/Myd88*^*fl/fl*^ mice, we sought to determine which immune subsets may be responsible for the improved outcomes in *Lyz2-Cre/Myd88*^*fl/fl*^ mice. We therefore depleted select immune populations to determine whether its loss would reverse improved survival. We found that mice depleted of granulocytes with αLy6G had similar outcomes compared to control mice (Supplemental Fig. S7B). Similarly, macrophage depletion with αCSF1R with or without clodronate liposomes also did not significantly affect outcomes (Supplemental Fig. S7C,D). These negative results are potentially due to pathway redundancy between cell types, or instead due to incomplete depletion of macrophage/monocyte populations observed with these techniques^56^. Because improved type I IFN responses were observed in multiple cell types in *Lyz2-Cre/Myd88*^*fl/fl*^ mice, we wanted to determine whether the improved response to RT in these mice was dependent on type I IFN. We therefore treated mice bearing PK5L1940 tumors with a blocking antibody to IFNAR1 5d prior to RT, with repeat blockade every 5d such that IFNAR1 signaling was blocked for 2 weeks post-RT. This resulted in a complete reversal of improved outcomes observed in *Lyz2-Cre/Myd88*^*fl/fl*^ mice (Fig. 6D). Altogether, these data indicate that MyD88 loss in myeloid cells permits a more positive immune environment following radiation, associated with de-repression of IFN responses and improved CD8^+^ T cell control.

## Discussion

Radiation therapy is often described as a vaccination event whereby new CD8^+^ cell responses are unleashed due to antigen and adjuvant release from dying cancer cells^55^. While TLR/MyD88 signaling has been shown to be critical for Batf3^+^ cDC1 cross-presentation to CD8^+^ T cells, we surprisingly found that MyD88 signaling was dispensable in *Itgax*-expressing cells for the response to radiation therapy but is instead highly impacted by myeloid MyD88 expression in pancreatic cancer. One potential explanation for this response is that TRIF/IRF signaling remained intact in myeloid cells in *Lyz2-Cre/Myd88*^*fl/fl*^ mice but the MyD88/NF-κB pathway was suppressed. This led to a decrease in expression of M2/Th2-type factors and increased expression of genes associated with type I and type II IFN signaling, which improved immune control dependent on type I IFN and CD8^+^ T cells. In contrast, *Itgax-Cre/Myd88*^*fl/fl*^ mice retained MyD88 signaling in TAM, monocyte, and granulocyte populations, thereby retaining myeloid-derived immunosuppression, and likely suppressed IFN responses.

We have previously shown that RT induces *Nfkb1* (NF-κB p105/p50) expression in macrophages that is detrimental to the response to RT^57,58^. This is potentially due in part to the increased propensity to form p50 homodimers that drive IL-10 production instead of p50:p65 heterodimers that drive *Il12b* expression^59,60^ or other dimer combinations that drive inflammatory gene expression^61,62^. This may also be associated with myeloid reprogramming that occurs following the interaction of macrophages with dying cancer cells that drives immunosuppression or tolerance^58,63^. Whether through these or additional mechanisms, it remains evident from our experiments that myeloid MyD88 signaling restricts production of type I interferon in response to RT, thereby limiting adaptive immune control of tumors in pancreatic cancer. This response was consistent across two cell lines, although the degree of improvement was different between them. Panc02-SIY cells are derived from a chemically induced model, where a Smad4 mutation is the only mutation commonly observed in human pancreatic cancer^64^. PK5L1940 cells were derived from KPC-LSIY mice, which have significantly fewer mutations, but carry the commonly observed *Kras* and *Tp53* mutations. The degree of difference is potentially due to the fact that the chemically induced Panc02-SIY cells carry more neoantigens against which T cells can be generated, though our experiments do not directly test this hypothesis. While the Cre-driven conditional knockouts limit our ability to treatment directly in Cre-driven spontaneous tumor models, additional studies to confirm the impact of ongoing and treatment-related MyD88-driven signaling in pancreatic cancer on outcome are needed to evaluate this in a more authentic tumor environment. However, our scRNASeq analysis of TLR and MyD88 expression in the PK5L1940 tumors derived from KPC-LSIY mice and patient pancreatic tumors suggest similar cells and pathways are involved in both settings.

Multiple cell types within the tumor microenvironment express MyD88 and its associated upstream receptors. While previous studies have shown that MyD88 expression in DCs is important for maturation and cross-presentation to T cells^21^, our results above indicate that MyD88 expression in tumor-associated myeloid cells suppress CD8^+^ T cell responses to RT dependent on type I IFN. Whether or not this response is due differential presence of NFκB subunits or other factors remains to be determined. On the other hand, MyD88 signaling is also important for antigen-specific T cell responses. Loss of MyD88 in T cells results in decreased clonal expansion and effector function in mice infected with LCMV^45,46^. Similarly, in mixed bone marrow chimeras, *Myd88*^*−/−*^ CD4^+^ and CD8^+^ T cells expanded less and had reduced IFNγ production than their WT counterparts in response to *T. cruzi* infection^51^. Similar results were observed in CD4^+^ T cells with transfer of *Myd88*^*−/−*^ splenocytes into *Rag1*^*−/−*^ mice^51^. While certainly important for primary expansion of CD8^+^ T cells after TCR engagement, MyD88 is not required for the maintenance of memory T cells as demonstrated by an inducible model of Myd88 deletion^46^. Altogether, these data indicate that a blockade of TLR/MyD88 signaling after antigen recognition and memory T cell formation might be beneficial by dampening myeloid-derived immunosuppression.

Type I IFN responses are associated with improved outcomes across various cancer pathologies and strategies to drive these responses are underway^65,66^. We and others have shown that combination of TLR3 agonists that do not signal through MyD88 are effective when combined with RT^54,67–69^, and other combinatorial strategies utilizing TLR7 or TLR9 agonists that also preferentially drive type I IFN responses also have improved response to RT in various cancers^70–74^. However, TLR9 has also been shown to limit response to RT in some instances^75^, while other studies have shown that targeting multiple TLRs with Salmonella typhimurium secreting flagellin B results in improved tumor suppression accompanied by an M2–M1 shift in macrophage polarization^76^. ^77^Together, these studies underscore the contextual nature of the response to TLR agonists or adjuvants released by dying cells, which may be dependent on dominant mechanisms of immune escape. Clinical trials utilizing TLR agonists in several solid tumors are currently underway, including in combination with radiation therapy and/or immunotherapy in pancreatic cancer. Our results indicate that the MyD88 pathway in myeloid cells may limit RT-mediated immune control of pancreatic cancer and agonists avoiding inflammatory MyD88 signaling or myeloid-specific MyD88 inhibition may be alternative strategies to improve outcomes.

## Methods

### Resource availability

#### Materials availability

Further information and resource requests should be directed to the Lead Contact, Michael Gough (Michael.gough@providence.org).

#### Data and code availability

scRNAseq and Nanostring data have been deposited into the GEO under accession number GSE176015. Data are accessible with reviewer code anahkgwsxferred.

### Experimental model and subject details

#### Animal studies and cell lines

All animal experiments were performed in compliance with the National Institutes of Health guidelines and were approved by the Institutional Animal Care and Use Committee at Earle A. Chiles Research Institute. All experiments were performed in accordance with relevant guidelines and regulations and is reported in accordance with ARRIVE guidelines. Euthanasia was performed by gradual CO_2_ asphyxiation and cervical dislocation according to AVMA guiedelines. Male and female experimental mice were used between 6 and 12 weeks of age, the number of mice per experimental group indicated in each figure and/or figure legend. Unblinded mice were randomized to treatment groups by cage with post hoc analysis of tumor volumes at time of RT performed to ensure tumor volumes were not significantly different at time of treatment across treatment groups. All animal experiments were performed a minimum of two times and pooled, unless otherwise noted.

*Myd88*^*−/−*^ (Stock #009,088)^23^, *Itgax-Cre* (CD11c; Stock #007,567)^77^, *Lck-Cre* (Stock #003,802)^78^, *Lyz2-Cre* (Stock #004,781)^79^, and *Myd88*^*fl/fl*^ (Stock #008,888)^23^ mice were purchased from Jackson Laboratory (Bar Harbor, ME). To generate mice with lineage-specific deletion of *Myd88*, we crossed *MyD88*^*fl/fl*^ mice with *Itgax-Cre* (CD11c-Cre), *Lck-Cre*, and *Lyz2-Cre* (LysM-Cre) mice to homozygosity, respectively.

Panc02-SIY cells were kindly provided by Dr. Weichselbaum (University of Chicago)^80^. PK5L1940 cells have been previously described^81^. Cells were cultured in RPMI 1640 supplemented with 10% FBS, 10 mM HEPES, 1 × non-essential amino acid, 110 μg/mL sodium pyruvate, 100 U/mL penicillin, 100 μg/mL streptomycin, and 55 μM β-mercaptoethanol. Panc02-SIY or PK5L1940 cells were suspended in PBS and injected subcutaneously at a concentration of 5.0 × 10^6^ or 0.2 × 10^6^ cells in 50μL, respectively. Tumor-bearing mice were monitored three times per week until tumors exceed 12 mm in size, or when body condition score declined. Tumor volume (V) was calculated using the formula V (mm^3^) = A x B^2^/2, where A reflected the larger tumor diameter and B the smaller diameter.

CT-guided radiation therapy was delivered using the XStrahl Small Animal Radiation Research Platform (Suwanee, GA)^82^, using a 10 × 10 mm collimator with beam angles designed to minimize normal tissue and draining lymph node exposure. Dosimetry was performed using MuriSlice software^83^, with treatment calculated to the tumor isocenter. All animals received 16 Gy RT 14 days post-implantation, when tumors were approximately 50–100 mm^3^.

In vivo blockade and depletion studies were performed by i.p. administration of αCD8 (2.43), αCSF1R (AFS98), αLy6G (1A8), or αIFNAR1 (MAR1-5A3) antibodies, all obtained from BioXcell (Branford, CT). 100 μg αCD8 was administered 5d prior to RT and every 7d thereafter for a total of 3 injections. 500 μg αCSF1R was given 5d prior to RT and 250 μg was given every 5d thereafter for a total of 6 doses. 1.0 mg of clodronate or control liposomes (Liposoma BV, Amsterdam, Netherlands) were administered concurrently with αCSF1R. 200 μg αLy6G was administered 1d prior to RT and 100 μg administered every 3-4d thereafter for a total of 7 injections. 250 μg of αIFNAR1 mAb was given 5d prior to RT and was given every 5d thereafter for a total of 4 doses.

Vaccination studies were performed by i.v. injection of 10^7^ *∆ActA-Ova Listeria monocytogenes*, followed by a boost 21 or 28 days later. 5d post-boost, mice were euthanized, and spleens were collected and prepared as described below for flow cytometry. For cytokine analyses, 10^6^ splenocytes were plated and stimulated with 2 mM SL8 peptides in the presence of brefeldin A for 4 h and stored overnight at 4° C before staining and data acquisition.

### Method details

#### Bone marrow-derived macrophages

Bone marrow-derived macrophages (BMMΦs) were generated from bone marrow isolated from femurs and tibias of MyD88^fl/fl^ or Lyz2-Cre/MyD88^fl/fl^ mice. Marrow was cultured in RPMI 1640 supplemented with 10% FBS, 10 mM HEPES, 1 × non-essential amino acid, 110 μg/mL sodium pyruvate, 100 U/mL penicillin, 100 μg/mL streptomycin, 55 μM β-mercaptoethanol, and 20 ng/mL recombinant murine CSF-1 (PeproTech or Invitrogen), and media was replaced every 3–4 days. On day 6–7, BMMΦs were stimulated with 100 ng/mL LPS for 24 h before supernatant was collected and cytokines were analyzed by ProcartaPlex (ThermoFisher Scientific) according to the manufacturer’s instructions.

#### Flow cytometry

Single cell suspensions were prepared from tumors, spleens, or blood. Tumors were manually minced using scissors, followed by a 45-min enzymatic digestion in HBSS supplemented with 250 U/mL collagenase IV (Worthington) and 30 U/mL DNaseI (Roche), 5 mM CaCl_2_, and 5% FBS at 37° C with constant agitation. Spleens were prepared by passing through a 70 μm nylon mesh strainers. Erythrocytes in spleens and blood were lysed using ACK lysing buffer (Gibco) according to the manufacturer’s instructions.

To prevent non-specific binding, cells were incubated for 30 min at 4° C with rat anti-mouse CD16/CD32 (1:500, BD Biosciences) in PBS containing Live/Dead Aqua stain (1:1000, Invitrogen) to stain for viable cells. Subsequently, cells were incubated in PBS containing 2 mM EDTA and 2% BSA for 30 min with 100 μL of fluorophore conjugated anti-mouse antibodies; CD45 (30-F11, BD Biosciences), CD3 (17A2, eBioscience) , CD8a (53–6.7, eBioscience), CD4 (RM5-5, eBioscience), CD11b (M1/70, BioLegend), CD11c (N418, BioLegend), Ly6G (1A8, BioLegend), Ly6C (HK1.4, BioLegend), F4/80 (BM8, BioLegend), NK1.1 (PK136, BioLegend), MHCII (M5/114.15.2, BioLegend), CD19 (6D5, BioLegend), CD44 (IM7, BioLegend), CD62L (MEL-14, eBioscience), KLRG1 (2F1, eBioscience), CD127 (SB/199, BD Pharmingen), IFNγ (XMG1.2, eBioscience), TNFα (MP6-XT22, eBioscience), IL-2 (JES6-5H4, eBioscience), SIYRYYGL pentamer (1803, Proimmune), and SIINFEKL tetramer (NIH Tetramer Core Facility at Emory University). Cells were then washed in PBS/EDTA/FBS, followed by a 15-min incubation in Fixation buffer or instead in Fix/Perm buffer for intracellular cytokine staining (ICS). For ICS studies, antibodies were diluted in PBS/EDTA/FBS containing permeabilization buffer and incubated for 30 min at 4° C. After a final wash, cells were resuspended in PBS/EDTA/BSA, and stored at 4° C until data acquisition on a BDLSRII using FACSDiva software (BD Biosciences)^84^. Analysis was performed using FlowJo software (Tree Star Inc.)^85^.

#### Nanostring

10–20 mg of snap frozen tumors were crushed on liquid nitrogen and RNA was extracted using the RNeasy Micro Kit (QIAGEN) according to the manufacturer’s instructions. Samples were then hybridized with NanoString pan-cancer immune profiling probes for 16 h and were subsequently loaded into an nCounter FLEX cartridge. Raw data were normalized utilizing default settings. Fold changes in gene expression were exported to Ingenuity Pathway Analysis (QIAGEN)^86^ for further analysis. Data were deposited to the NCBI GEO under accession number GSE176014.

#### Single cell RNA sequencing

Single cell suspensions from PK5L1940 tumors 3d post-RT (16 Gy) were prepared as described above. Immune cells from single cell suspensions were positively selected using anti-mouse CD45 MicroBeads (Miltenyi Biotec). Cells were then incubated in PBS containing Live/Dead Aqua as described above, before a secondary incubation with CD45. Live CD45^+^ cells were sorted using a BD FACSAria. Cells were processed according to the manufacturers protocol for the Chromium Single Cell 3’ Reagent kit (v3.0) from 10X Genomics. Libraries were sequenced using an Illumina NovaSeq 6000 using a NovaSeq 6000 S2 reagent kit (v1.0). Data were processed using the Cell Ranger pipeline (v3.1) and subsequently analyzed with the Loupe Browser from 10X Genomics (v5.0)^87^. Using the Loupe Browser differentially expressed genes between groups were considered significant if the gene expression fold change was > 1.5 and the Benjamini–Hochberg adjusted *p* value was < 0.1. Volcano plots were generated using the EnhancedVolcano package (v1.7.16) in R (v4.0.2). Additional analyses were performed with Ingenuity Pathway Analysis (IPA) software from Qiagen (v01-19-00)^86^ using default settings for Core Analysis, with expression of *Lyz2*, and the sex-specific genes *Ddx3y* and *Xist* excluded from downstream analyses. Data were deposited to the NCBI GEO under accession number GSE176011.

Raw human scRNA seq data were acquired from GSE212966. Data were processed using the latest stable Cell Ranger pipeline (v7.1) and subsequently analyzed with the Loupe Browser from 10X Genomics (v6.4.1)^87^. Relative gene expression was assessed on *PTPRC*^+^ (CD45^+^) immune cells in PDAC samples only, with all non-immune cells excluded from analysis.

#### Quantification and statistical analysis

Statistical analyses were performed using Prism 6^88^ for Mac. Data points represent biological replicates and are represented as mean + /− SEM unless otherwise indicated. Specific tests included unpaired two-tailed t test, one-way ANOVA, and log-rank. In instances where multiple comparisons were performed, Tukey correction was used, unless otherwise specified. Specific tests are identified in the respective figures. *p* values < 0.05 were considered statistically significant, with * *p* < 0.05, ** *p* < 0.01, *** *p* < 0.001, **** *p* < 0.0001, unless otherwise indicated in the figure. Heat maps were generated using ClustVis (https://biit.cs.ut.ee/clustvis/), with rows centered and unit variance scaling applied to rows. Both rows and columns were clustered using Euclidean distance and average linkage. Volcano plots were generated using the EnhancedVolcano package (v1.7.16) in R (v4.0.2). Additional upstream molecule pathway analysis was performed with Ingenuity Pathway Analysis (IPA) software from Qiagen (v01-19-00)^86^ using default settings for Core Analysis.

## Supplementary Information

Supplementary Figures.

## References

[1] Medler T (2019). Activating the nucleic acid-sensing machinery for anticancer immunity. Int. Rev. Cell Mol. Biol..

[2] Garg AD (2015). Molecular and translational classifications of DAMPs in immunogenic cell death. Front. Immunol..

[3] Pichler M (2012). Histologic tumor necrosis is an independent prognostic indicator for clear cell and papillary renal cell carcinoma. Am. J. Clin. Pathol..

[4] Pollheimer MJ (2010). Tumor necrosis is a new promising prognostic factor in colorectal cancer. Hum. Pathol..

[5] Atanasov G (2017). Tumor necrosis and infiltrating macrophages predict survival after curative resection for cholangiocarcinoma. Oncoimmunology.

[6] Maiorano E (2010). Prognostic and predictive impact of central necrosis and fibrosis in early breast cancer: Results from two international breast cancer study group randomized trials of chemoendocrine adjuvant therapy. Breast Cancer Res. Treat..

[7] Gkogkou C, Frangia K, Saif MW, Trigidou R, Syrigos K (2014). Necrosis and apoptotic index as prognostic factors in non-small cell lung carcinoma: A review. Springerplus.

[8] Richards CH, Mohammed Z, Qayyum T, Horgan PG, McMillan DC (2011). The prognostic value of histological tumor necrosis in solid organ malignant disease: A systematic review. Future Oncol..

[9] Chao MP (2010). Calreticulin is the dominant pro-phagocytic signal on multiple human cancers and is counterbalanced by CD47. Sci. Transl. Med..

[10] Wu T (2016). HMGB1 overexpression as a prognostic factor for survival in cancer: A meta-analysis and systematic review. Oncotarget.

[11] Hubert P (2021). Extracellular HMGB1 blockade inhibits tumor growth through profoundly remodeling immune microenvironment and enhances checkpoint inhibitor-based immunotherapy. J. Immunother. Cancer.

[12] Harding SM (2017). Mitotic progression following DNA damage enables pattern recognition within micronuclei. Nature.

[13] Vanpouille-Box C (2017). DNA exonuclease Trex1 regulates radiotherapy-induced tumour immunogenicity. Nat. Commun..

[14] Baird JR (2017). Stimulating innate immunity to enhance radiation therapy-induced tumor control. Int. J. Radiat. Oncol. Biol. Phys..

[15] Pena OM, Pistolic J, Raj D, Fjell CD, Hancock RE (2011). Endotoxin tolerance represents a distinctive state of alternative polarization (M2) in human mononuclear cells. J. Immunol..

[16] Mantovani A, Sica A, Locati M (2005). Macrophage polarization comes of age. Immunity.

[17] Mantovani A, Sozzani S, Locati M, Allavena P, Sica A (2002). Macrophage polarization: Tumor-associated macrophages as a paradigm for polarized M2 mononuclear phagocytes. Trends Immunol..

[18] Takeda K, Akira S (2004). TLR signaling pathways. Semin. Immunol..

[19] Kawai T, Akira S (2010). The role of pattern-recognition receptors in innate immunity: Update on Toll-like receptors. Nat. Immunol..

[20] Fitzgerald KA, Kagan JC (2020). Toll-like receptors and the control of immunity. Cell.

[21] Apetoh L (2007). Toll-like receptor 4-dependent contribution of the immune system to anticancer chemotherapy and radiotherapy. Nat. Med..

[22] Shi Y, Evans JE, Rock KL (2003). Molecular identification of a danger signal that alerts the immune system to dying cells. Nature.

[23] Hou B, Reizis B, DeFranco AL (2008). Toll-like receptors activate innate and adaptive immunity by using dendritic cell-intrinsic and -extrinsic mechanisms. Immunity.

[24] Engelhardt JJ (2012). Marginating dendritic cells of the tumor microenvironment cross-present tumor antigens and stably engage tumor-specific T cells. Cancer Cell.

[25] Fuertes MB (2011). Host type I IFN signals are required for antitumor CD8+ T cell responses through CD8{alpha}+ dendritic cells. J. Exp. Med..

[26] Krysko DV (2011). TLR-2 and TLR-9 are sensors of apoptosis in a mouse model of doxorubicin-induced acute inflammation. Cell Death Differ..

[27] Yamazaki T (2014). Defective immunogenic cell death of HMGB1-deficient tumors: Compensatory therapy with TLR4 agonists. Cell Death Differ..

[28] Tesniere A (2010). Immunogenic death of colon cancer cells treated with oxaliplatin. Oncogene.

[29] Apetoh L, Tesniere A, Ghiringhelli F, Kroemer G, Zitvogel L (2008). Molecular interactions between dying tumor cells and the innate immune system determine the efficacy of conventional anticancer therapies. Cancer Res..

[30] Asea A (2002). Novel signal transduction pathway utilized by extracellular HSP70: Role of toll-like receptor (TLR) 2 and TLR4. J. Biol. Chem..

[31] Sobek V (2004). Direct Toll-like receptor 2 mediated co-stimulation of T cells in the mouse system as a basis for chronic inflammatory joint disease. Arthritis Res. Ther..

[32] Tabiasco J (2006). Human effector CD8+ T lymphocytes express TLR3 as a functional coreceptor. J. Immunol..

[33] Newman AM (2019). Determining cell type abundance and expression from bulk tissues with digital cytometry. Nat. Biotechnol..

[34] Chen K (2022). Single cell RNA-seq identifies immune-related prognostic model and key signature-SPP1 in pancreatic ductal adenocarcinoma. Genes (Basel).

[35] Metsalu T, Vilo J (2015). ClustVis: A web tool for visualizing clustering of multivariate data using principal component analysis and heatmap. Nucleic Acids Res..

[36] Priebe TS, Atkinson EN, Pan BF, Nelson JA (1992). Intrinsic resistance to anticancer agents in the murine pancreatic adenocarcinoma PANC02. Cancer Chemother. Pharmacol..

[37] Medler TR (2023). Tumor resident memory CD8 T cells and concomitant tumor immunity develop independently of CD4 help. Sci. Rep..

[38] Deng L (2014). STING-dependent cytosolic DNA sensing promotes radiation-induced Type I interferon-dependent antitumor immunity in immunogenic tumors. Immunity.

[39] Nywening TM (2018). Targeting both tumour-associated CXCR2(+) neutrophils and CCR2(+) macrophages disrupts myeloid recruitment and improves chemotherapeutic responses in pancreatic ductal adenocarcinoma. Gut.

[40] Gajewski TF, Schreiber H, Fu YX (2013). Innate and adaptive immune cells in the tumor microenvironment. Nat. Immunol..

[41] Yu R, Zhu B, Chen D (2022). Type I interferon-mediated tumor immunity and its role in immunotherapy. Cell Mol. Life Sci..

[42] Geng D (2010). Amplifying TLR-MyD88 signals within tumor-specific T cells enhances antitumor activity to suboptimal levels of weakly immunogenic tumor antigens. Cancer Res..

[43] Sanchez-Ruiz M (2019). TLR signals license CD8 T cells to destroy oligodendrocytes expressing an antigen shared with a Listeria pathogen. Eur. J. Immunol..

[44] Hu Z (2014). Boosting functional avidity of CD8+ T cells by vaccinia virus vaccination depends on intrinsic T-cell MyD88 expression but not the inflammatory milieu. J. Virol..

[45] Rahman AH (2008). MyD88 plays a critical T cell-intrinsic role in supporting CD8 T cell expansion during acute lymphocytic choriomeningitis virus infection. J. Immunol..

[46] Rahman AH (2011). Antiviral memory CD8 T-cell differentiation, maintenance, and secondary expansion occur independently of MyD88. Blood.

[47] Gelman AE (2006). The adaptor molecule MyD88 activates PI-3 kinase signaling in CD4+ T cells and enables CpG oligodeoxynucleotide-mediated costimulation. Immunity.

[48] Frazer LC (2013). CD4+ T cell expression of MyD88 is essential for normal resolution of Chlamydia muridarum genital tract infection. J. Immunol..

[49] LaRosa DF (2008). T cell expression of MyD88 is required for resistance to Toxoplasma gondii. Proc. Natl. Acad. Sci. USA.

[50] Zhou S (2009). MyD88 intrinsically regulates CD4 T-cell responses. J. Virol..

[51] Oliveira A-C (2017). Crucial role for T cell-intrinsic IL-18R-MyD88 signaling in cognate immune response to intracellular parasite infection. Elife.

[52] Quigley M, Martinez J, Huang X, Yang Y (2009). A critical role for direct TLR2-MyD88 signaling in CD8 T-cell clonal expansion and memory formation following vaccinia viral infection. Blood.

[53] Schenten D (2014). Signaling through the adaptor molecule MyD88 in CD4+ T cells is required to overcome suppression by regulatory T cells. Immunity.

[54] Blair TC (2020). Dendritic cell maturation defines immunological responsiveness of tumors to radiation therapy. J. Immunol..

[55] Medler TR, Blair TC, Crittenden MR, Gough MJ (2021). Defining immunogenic and radioimmunogenic tumors. Front. Oncol..

[56] Zhu Y (2017). Tissue-resident macrophages in pancreatic ductal adenocarcinoma originate from embryonic hematopoiesis and promote tumor progression. Immunity.

[57] Crittenden MR (2012). Expression of NF-kappaB p50 in tumor stroma limits the control of tumors by radiation therapy. PLoS ONE.

[58] Crittenden MR (2016). Mertk on tumor macrophages is a therapeutic target to prevent tumor recurrence following radiation therapy. Oncotarget.

[59] Cao S, Zhang X, Edwards JP, Mosser DM (2006). NF-kappaB1 (p50) homodimers differentially regulate pro- and anti-inflammatory cytokines in macrophages. J. Biol. Chem..

[60] Ishizuka EK, Filgueiras LR, Rios FJ, Serezani CH, Jancar S (2016). PAFR activation of NF-kappaB p65 or p105 precursor dictates pro- and anti-inflammatory responses during TLR activation in murine macrophages. Sci. Rep..

[61] Siggers T (2011). Principles of dimer-specific gene regulation revealed by a comprehensive characterization of NF-kappaB family DNA binding. Nat. Immunol..

[62] Zhang Q, Lenardo MJ, Baltimore D (2017). 30 years of NF-kappaB: A blossoming of relevance to human pathobiology. Cell.

[63] Wallet MA (2008). MerTK is required for apoptotic cell-induced T cell tolerance. J. Exp. Med..

[64] Wang Y (2012). Genomic sequencing of key genes in mouse pancreatic cancer cells. Curr. Mol. Med..

[65] Medrano RFV, Hunger A, Mendonca SA, Barbuto JAM, Strauss BE (2017). Immunomodulatory and antitumor effects of type I interferons and their application in cancer therapy. Oncotarget.

[66] Zitvogel L, Galluzzi L, Kepp O, Smyth MJ, Kroemer G (2015). Type I interferons in anticancer immunity. Nat. Rev. Immunol..

[67] Hammerich L (2019). Systemic clinical tumor regressions and potentiation of PD1 blockade with in situ vaccination. Nat. Med..

[68] Le UM, Kaurin DG, Sloat BR, Yanasarn N, Cui Z (2009). Localized irradiation of tumors prior to synthetic dsRNA therapy enhanced the resultant anti-tumor activity. Radiother. Oncol..

[69] Yoshida S (2018). Toll-like receptor 3 signal augments radiation-induced tumor growth retardation in a murine model. Cancer Sci..

[70] Dewan MZ (2012). Synergy of topical toll-like receptor 7 agonist with radiation and low-dose cyclophosphamide in a mouse model of cutaneous breast cancer. Clin. Cancer Res..

[71] Mason KA (2005). Targeting toll-like receptor 9 with CpG oligodeoxynucleotides enhances tumor response to fractionated radiotherapy. Clin. Cancer Res..

[72] Milas L (2004). CpG oligodeoxynucleotide enhances tumor response to radiation. Cancer Res..

[73] Meng Y (2005). Successful combination of local CpG-ODN and radiotherapy in malignant glioma. Int. J. Cancer.

[74] Zhang H (2012). An in situ autologous tumor vaccination with combined radiation therapy and TLR9 agonist therapy. PLoS ONE.

[75] Gao C (2013). TLR9 signaling in the tumor microenvironment initiates cancer recurrence after radiotherapy. Cancer Res..

[76] Zheng JH (2017). Two-step enhanced cancer immunotherapy with engineered Salmonella typhimurium secreting heterologous flagellin. Sci. Transl. Med..

[77] Stranges PB (2007). Elimination of antigen-presenting cells and autoreactive T cells by Fas contributes to prevention of autoimmunity. Immunity.

[78] Hennet T, Hagen FK, Tabak LA, Marth JD (1995). T-cell-specific deletion of a polypeptide N-acetylgalactosaminyl-transferase gene by site-directed recombination. Proc. Natl. Acad. Sci. USA.

[79] Clausen BE, Burkhardt C, Reith W, Renkawitz R, Forster I (1999). Conditional gene targeting in macrophages and granulocytes using LysMcre mice. Transgenic Res..

[80] Zheng W (2016). Combination of radiotherapy and vaccination overcomes checkpoint blockade resistance. Oncotarget.

[81] Crittenden MR (2018). Tumor cure by radiation therapy and checkpoint inhibitors depends on pre-existing immunity. Sci. Rep..

[82] SarrpCS v. 4.3.0 (Xstrahl, Suwanee, GA).

[83] MuriSlice v. 2.1.2 (Xstrahl, Suwanee, GA).

[84] BD FACSDiva v. 8.0 (Becton Dickinson & Company).

[85] FlowJo v. 10.8.1 (Becton Dickinson & Company).

[86] Ingenuity Pathway Analysis v. 01-19-00 (Qiagen).

[87] Loupe Browser v. 5.0 and 6.4.1 (10x Genomics).

[88] Prism v. 86.4.1 (GraphPad Software, Boston, MA).

