# Supplementary material for: Myeloid MyD88 restricts CD8+ T cell response to radiation therapy in pancreatic cancer
Source: Sci Rep. 2023 May 27;13:8634. doi: 10.1038/s41598-023-35834-w (PMC10224952; doi:10.1038/s41598-023-35834-w)
Supplement: Supplementary file 1 — Supplementary Figures. [file 41598_2023_35834_MOESM1_ESM.pdf]

SUPPLEMENTAL INFORMATION

Myeloid MyD88 restricts CD8$^+$ T cell response to radiation therapy in pancreatic cancer

Terry R. Medler[1], Tiffany C. Blair[1,2], Alejandro F. Alice[1], Alexa K. Dowdell[1], Brian D. Piening[1], Marka R. Crittenden[1,3], Michael J. Gough[1,*]

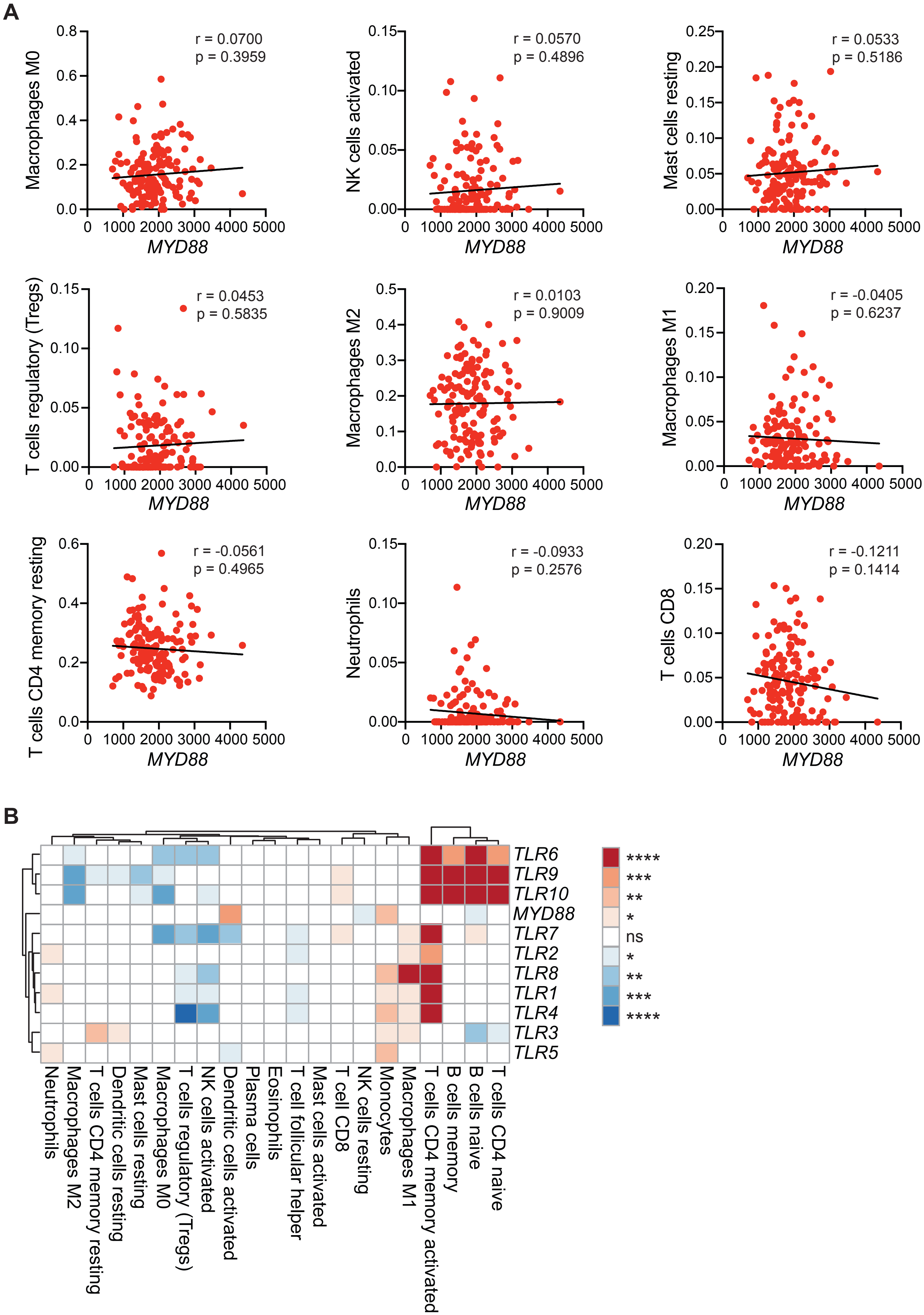
A
r = 0.0700
p = 0.3959
Macrophages M0
MYD88
r = 0.0570
p = 0.4896
NK cells activated
MYD88
r = 0.0533
p = 0.5186
Mast cells resting
MYD88
r = 0.0453
p = 0.5835
T cells regulatory (Tregs)
MYD88
r = 0.0103
p = 0.9009
Macrophages M2
MYD88
r = -0.0405
p = 0.6237
Macrophages M1
MYD88
r = -0.0561
p = 0.4965
T cells CD4 memory resting
MYD88
r = -0.0933
p = 0.2576
Neutrophils
MYD88
r = -0.1211
p = 0.1414
T cells CD8
MYD88
B
TLR6
TLR9
TLR10
MYD88
TLR7
TLR2
TLR8
TLR1
TLR4
TLR3
TLR5
****
***
**
*
ns
*
**
***
****
Neutrophils
Macrophages M2
T cells CD4 memory resting
Dendritic cells resting
Mast cells resting
Macrophages M0
T cells regulatory (Tregs)
NK cells activated
Dendritic cells activated
Plasma cells
Eosinophils
T cell follicular helper
Mast cells activated
T cell CD8
NK cells resting
Monocytes
Macrophages M1
T cells CD4 memory activated
B cells memory
B cells naive
T cells CD4 naive

Supplemental Figure S1. ***MYD88*** **and** ***TLR*** **expression in human pancreatic cancer.**

(A) Linear regression analysis showing Pearson correlation between MYD88 expression and inferred presence of indicated immune subsets as estimated by CIBERSORT in the PAAD TCGA dataset (n = 149). **(B)** Heatmap of p values for linear regression analysis of TLR and MYD88 expression with presence of immune subsets as inferred by CIBERSORT in the PAAD TCGA dataset (n = 149). Positive p values are indicated in shades of red, while negative p vaues appear in blue. Data were visualized using ClustVis. See **Figure 1C** for Pearson correlation values.

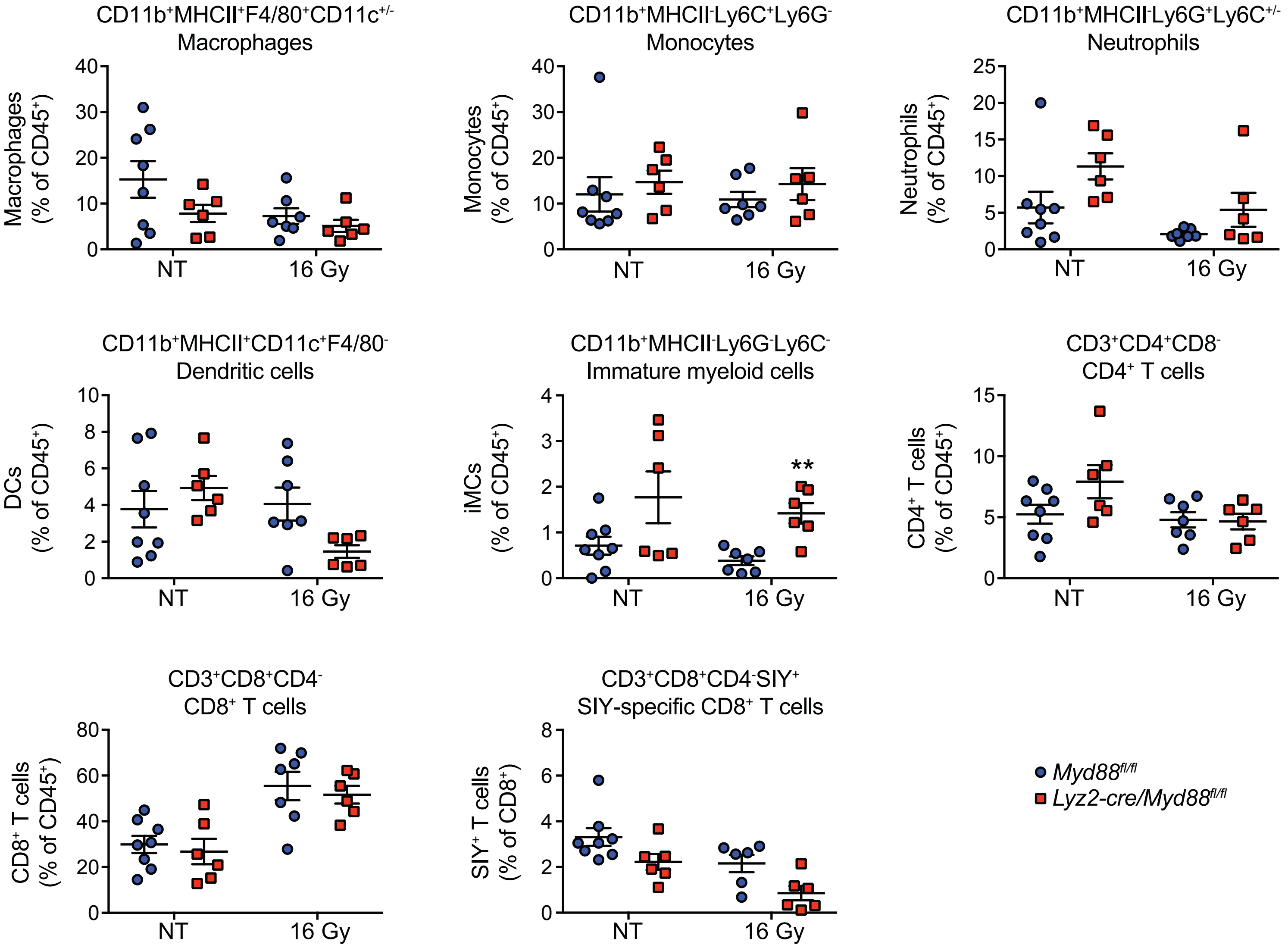

CD11b+MHCII+F4/80+CD11c+/-
Macrophages
Macrophages (% of CD45+)
0 10 20 30 40
NT
16 Gy
CD11b+MHCII-Ly6C+Ly6G-
Monocytes
Monocytes (% of CD45+)
0 10 20 30 40
NT
16 Gy
CD11b+MHCII-Ly6G+Ly6C+/-
Neutrophils
Neutrophils (% of CD45+)
0 5 10 15 20 25
NT
16 Gy
CD11b+MHCII+CD11c+F4/80-
Dendritic cells
DCs (% of CD45+)
0 2 4 6 8 10
NT
16 Gy
CD11b+MHCII-Ly6G-Ly6C-
Immature myeloid cells
iMCs (% of CD45+)
0 1 2 3 4
**
NT
16 Gy
CD3+CD4+CD8-
CD4+ T cells
CD4+ T cells (% of CD45+)
0 5 10 15
NT
16 Gy
CD3+CD8+CD4-
CD8+ T cells
CD8+ T cells (% of CD45+)
0 20 40 60 80
NT
16 Gy
CD3+CD8+CD4-SIY+
SIY-specific CD8+ T cells
SIY+ T cells (% of CD8+)
0 2 4 6 8
NT
16 Gy
Myd88fl/fl
Lyz2-cre/Myd88fl/fl

Supplemental Figure S2. Infiltrating immune cells are largely unchanged in *Lyz2-Cre/Myd88$^{fl/fl}$* mice.

Flow cytometric analysis of Panc02-SIY tumors isolated 7d post-RT using the indicated markers, represented as a percentage of live $CD45^+$ cells. Each data point represents a single mouse (n = 6-8 mice per group). Significant differences between genotypes were determined by multiple t tests corrected using the Holm-Sidak method, with ** $p < 0.01$.

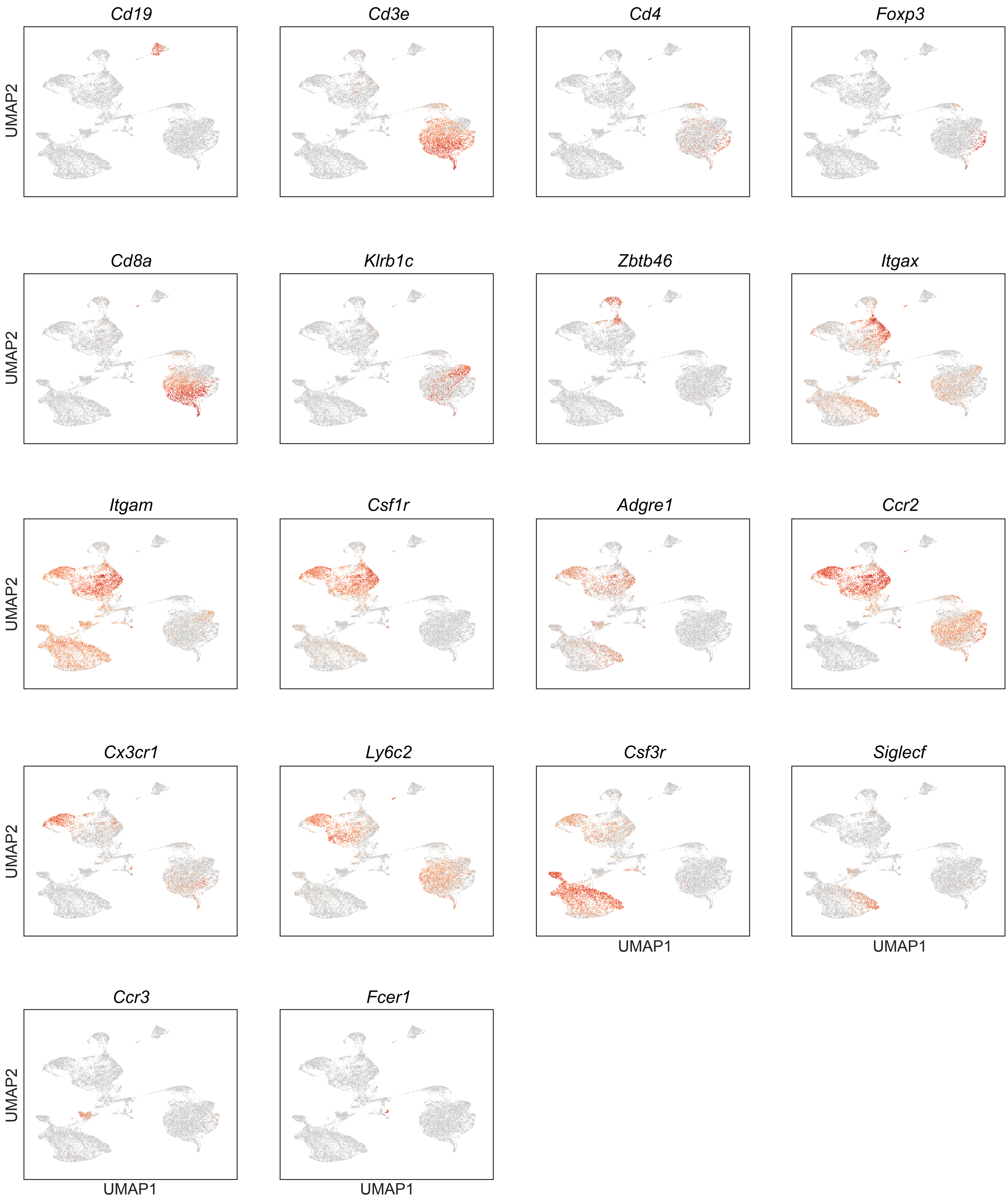
Cd19
Cd3e
Cd4
Foxp3
Cd8a
Klrb1c
Zbtb46
Itgax
Itgam
Csf1r
Adgre1
Ccr2
Cx3cr1
Ly6c2
Csf3r
Siglecf
Ccr3
Fcer1
UMAP2
UMAP1

Supplemental Figure S3. Expression of lineage markers used to identify dominant immune populations in scRNAseq.

scRNAseq was performed on $CD45^{+}$ cells isolated from untreated tumors or 3d post-RT in *Lyz2-Cre/Myd88*$^{fl/fl}$ mice and *Myd88*$^{fl/fl}$ mice. Expression of indicated genes in UMAP is shown (see also **Figure 3 and Supplemental Figure S4**), with the intensity of red directly proportional to the degree of expression.

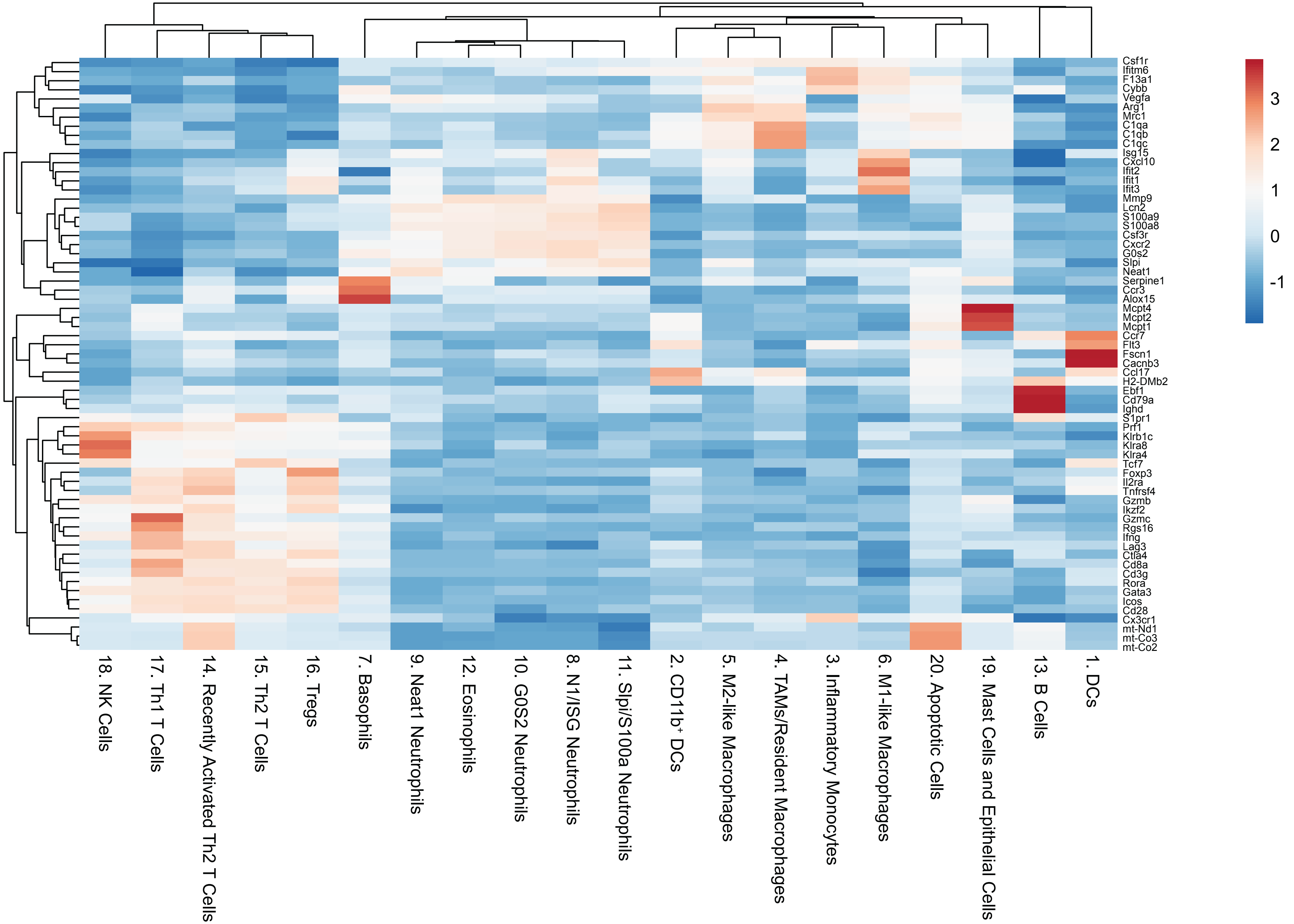

Csf1r
Ifitm6
F13a1
Cybb
Vegfa
Arg1
Mrc1
C1qa
C1qb
C1qc
Isg15
Cxcl10
Ifit2
Ifit1
Ifit3
Mmp9
Lcn2
S100a9
S100a8
Csf3r
Cxcr2
G0s2
Slpi
Neat1
Serpine1
Ccr3
Alox15
Mcpt4
Mcpt2
Mcpt1
Ccr7
Flt3
Fscn1
Cacnb3
Ccl17
H2-DMb2
Ebf1
Cd79a
Ighd
S1pr1
Prf1
Klrb1c
Klra8
Klra4
Tcf7
Foxp3
Il2ra
Tnfrsf4
Gzmb
Ikzf2
Gzmc
Rgs16
Ifng
Lag3
Ctla4
Cd8a
Cd3g
Rora
Gata3
Icos
Cd28
Cx3cr1
mt-Nd1
mt-Co3
mt-Co2
3
2
1
0
-1
18. NK Cells
17. Th1 T Cells
14. Recently Activated Th2 T Cells
15. Th2 T Cells
16. Tregs
7. Basophils
9. Neat1 Neutrophils
12. Eosinophils
10. G0S2 Neutrophils
8. N1/ISG Neutrophils
11. Slpi/S100a Neutrophils
2. CD11b+ DCs
5. M2-like Macrophages
4. TAMs/Resident Macrophages
3. Inflammatory Monocytes
6. M1-like Macrophages
20. Apoptotic Cells
19. Mast Cells and Epithelial Cells
13. B Cells
1. DCs

Supplemental Figure S4. Expression of lineage markers used to identify dominant immune populations in scRNAseq.

scRNAseq was performed on $CD45^+$ cells isolated from untreated tumors or 3d post-RT in *Lyz2-Cre/Myd88*$^{fl/fl}$ mice and *Myd88*$^{fl/fl}$ mice. Unsupervised hierarchical clustering of differential gene expression of indicated genes using ClustVis is shown (see also **Figure 3** and **Supplemental Figure S3**).

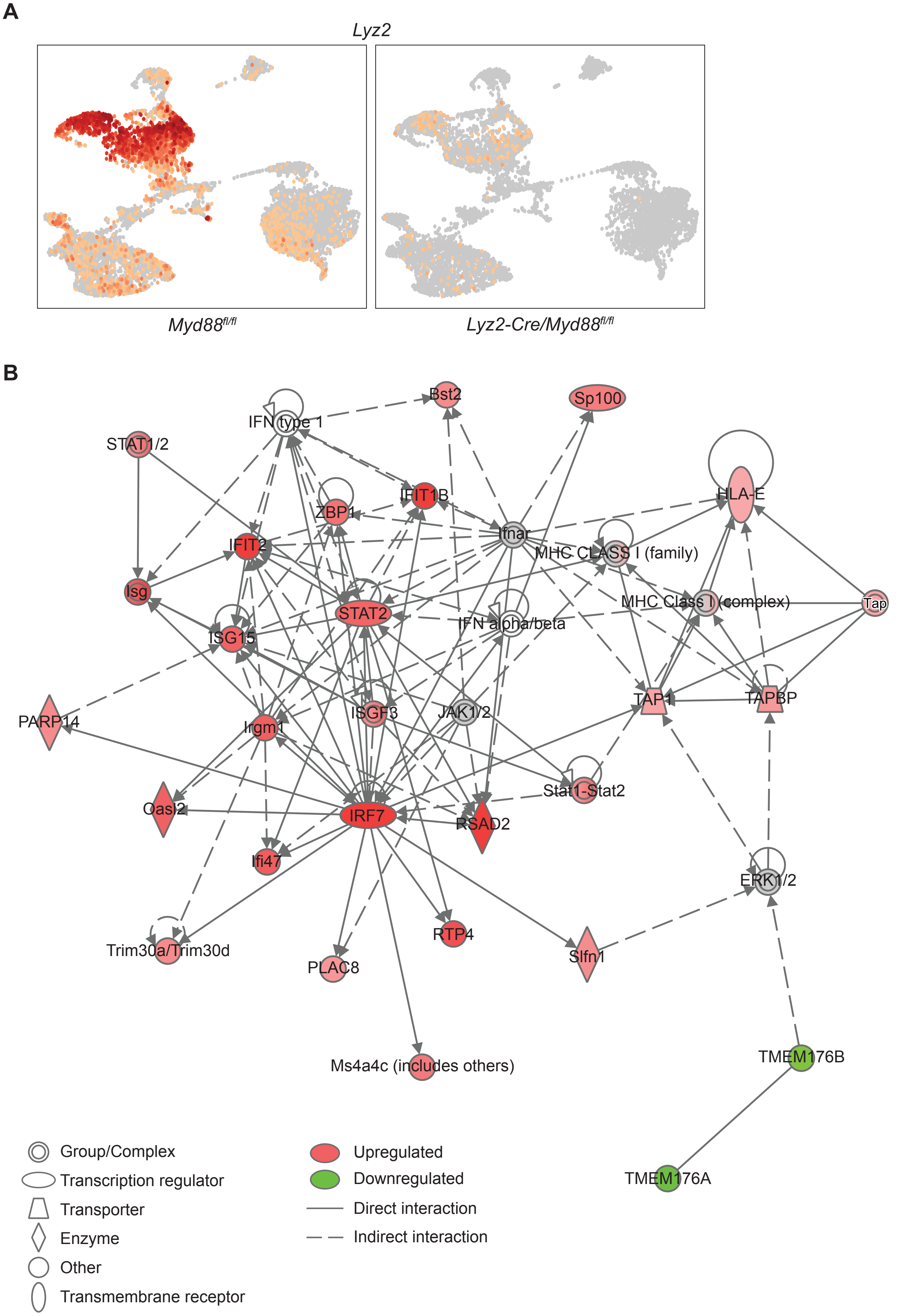
A
Lyz2
Myd88fl/fl
Lyz2-Cre/Myd88fl/fl
B
IFN type 1
Bst2
Sp100
STAT1/2
HLA-E
IFIT1B
ZBP1
Ifnar
IFIT2
MHC CLASS I (family)
Isg
MHC Class I (complex)
Tap
STAT2
IFN alpha/beta
ISG15
TAP1
TAPBP
PARP14
ISGF3
JAK1/2
Irgm1
Stat1-Stat2
Oasl2
IRF7
RSAD2
Ifi47
ERK1/2
RTP4
Trim30a/Trim30d
PLAC8
Slfn1
Ms4a4c (includes others)
TMEM176B
TMEM176A
Group/Complex
Transcription regulator
Transporter
Enzyme
Other
Transmembrane receptor
Upregulated
Downregulated
Direct interaction
Indirect interaction

Supplemental Figure S5. Enhanced interferon responses characterize infiltrating macrophages and monocytes in tumors from *Lyz2-Cre/Myd88$^{fl/fl}$* mice.

(A) UMAP of Lyz2 expression in *Lyz2-Cre/Myd88$^{fl/fl}$* mice and *Myd88$^{fl/fl}$* mice. **(B)** IPA Network analysis of significant differentially regulated genes in *Lyz2-Cre/Myd88$^{fl/fl}$* mice compared to *Myd88$^{fl/fl}$* mice. Genes upregulated in *Lyz2-Cre/Myd88$^{fl/fl}$* mice are depicted in red, while genes upregulated in *Myd88$^{fl/fl}$* mice are depicted in green, with color intensity associated with degree of upgregulation. Chosen network consists of the top scoring network (score = 52). Significance of differential gene expression was defined as fold change >1.5 and $p < 0.1$.

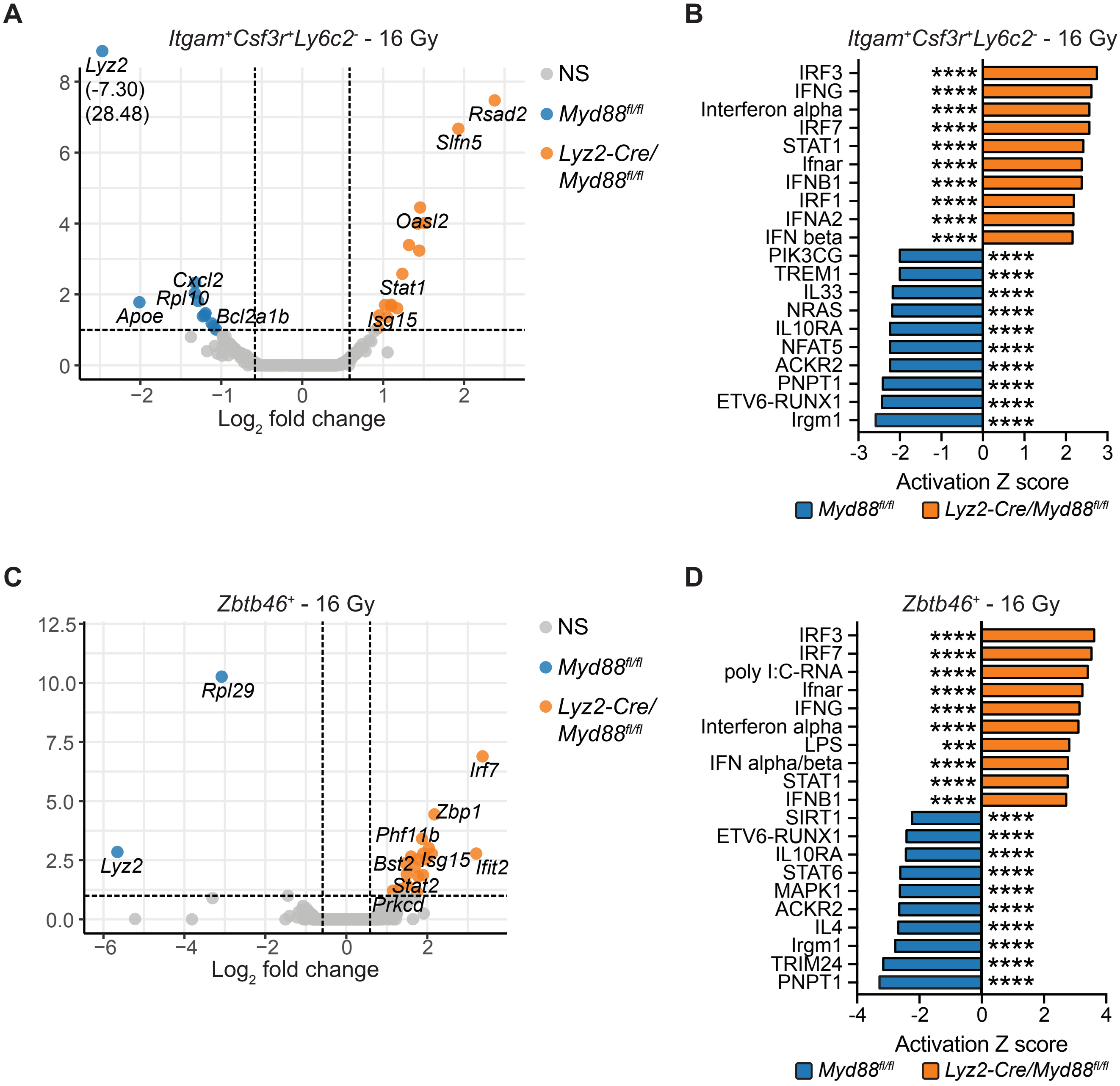
A
Itgam+Csf3r+Ly6c2- - 16 Gy
Lyz2
(-7.30)
(28.48)
Rsad2
Slfn5
Oasl2
Stat1
Isg15
Cxcl2
Rpl10
Apoe
Bcl2a1b
Log2 fold change
NS
Myd88fl/fl
Lyz2-Cre/
Myd88fl/fl
B
Itgam+Csf3r+Ly6c2- - 16 Gy
IRF3
IFNG
Interferon alpha
IRF7
STAT1
Ifnar
IFNB1
IRF1
IFNA2
IFN beta
PIK3CG
TREM1
IL33
NRAS
IL10RA
NFAT5
ACKR2
PNPT1
ETV6-RUNX1
Irgm1
Activation Z score
Myd88fl/fl
Lyz2-Cre/Myd88fl/fl
C
Zbtb46+ - 16 Gy
Rpl29
Irf7
Zbp1
Phf11b
Lyz2
Bst2
Isg15
Ifit2
Stat2
Prkcd
Log2 fold change
NS
Myd88fl/fl
Lyz2-Cre/
Myd88fl/fl
D
Zbtb46+ - 16 Gy
IRF3
IRF7
poly I:C-RNA
Ifnar
IFNG
Interferon alpha
LPS
IFN alpha/beta
STAT1
IFNB1
SIRT1
ETV6-RUNX1
IL10RA
STAT6
MAPK1
ACKR2
IL4
Irgm1
TRIM24
PNPT1
Activation Z score
Myd88fl/fl
Lyz2-Cre/Myd88fl/fl

Supplemental Fig. S6. Enhanced interferon responses characterize granulocytes and dendritic cells in tumors from *Lyz2-Cre/Myd88$^{fl/fl}$* mice.

(A) Volcano plot of differential gene expression in granulocytes (*Itgam^{+}Csf3r^{+}Ly6c2^{+}* cells) that are upregulated in *Lyz2-Cre/Myd88$^{fl/fl}$* mice (right, orange) or upregulated in *Myd88$^{fl/fl}$* mice (left, blue). **(B)** Upstream analysis *Itgam^{+}Csf3r^{+}Ly6c2^{+}* cells granulocytes using Ingenuity Pathway Analysis identifying likely regulators of increased differential gene expression observed in *Lyz2-Cre/Myd88$^{fl/fl}$* mice (top, orange) or *Myd88$^{fl/fl}$* mice (bottom, blue). **(C)** Volcano plot of differential gene expression in dendritic cells (*Zbtb46^{+}* cells) that are upregulated in *Lyz2-Cre/Myd88$^{fl/fl}$* mice (right, orange) or upregulated in *Myd88$^{fl/fl}$* mice (left, blue). **(D)** Upstream analysis *Zbtb46^{+}* using Ingenuity Pathway Analysis identifying likely regulators of increased differential gene expression observed in *Lyz2-Cre/Myd88$^{fl/fl}$* mice (top, orange) or *Myd88$^{fl/fl}$* mice (bottom, blue). Significance of differential gene expression in **(A-D)** was defined as fold change >1.5 and $p < 0.1$. **** $p < 0.0001$.

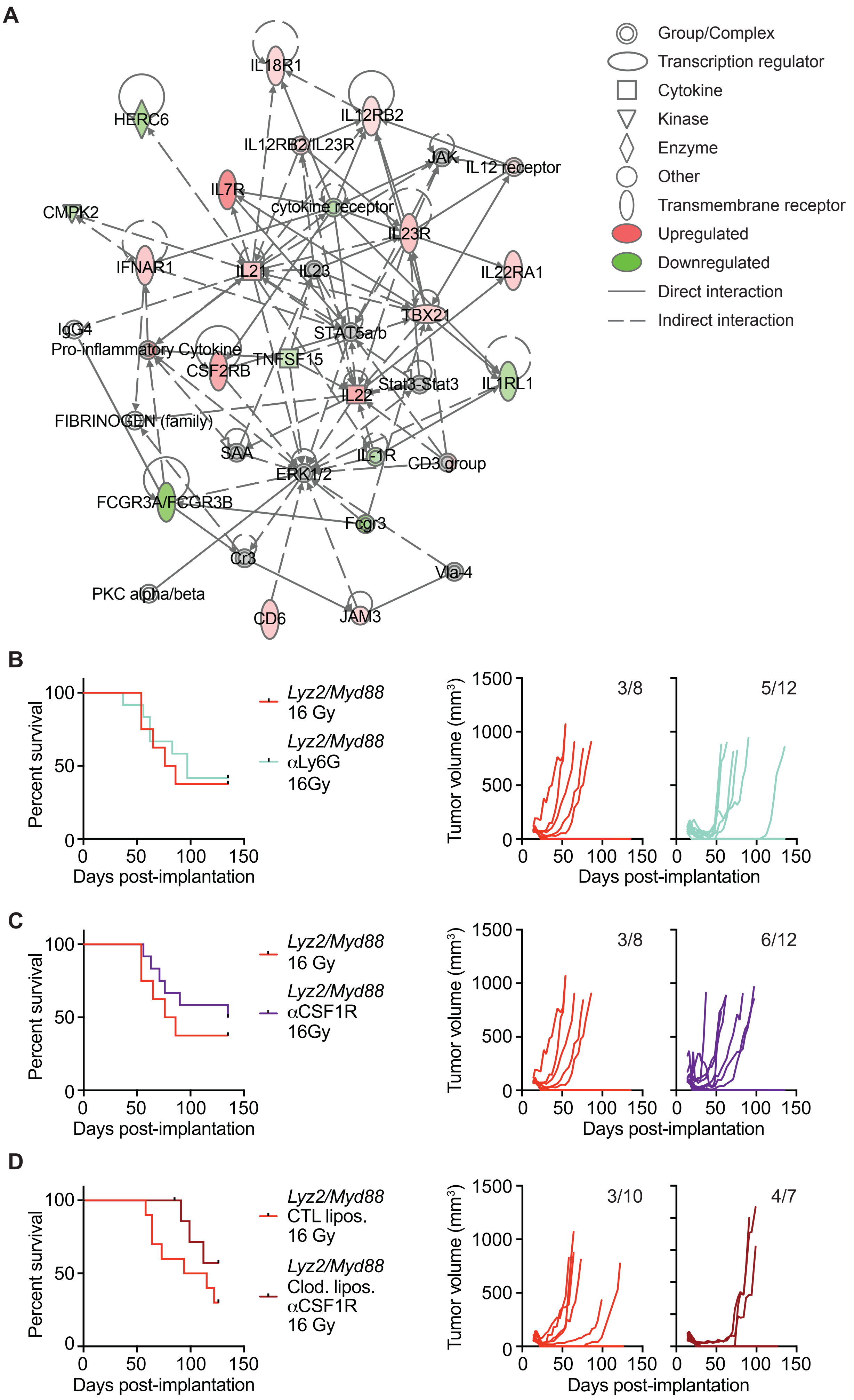
A
Group/Complex
Transcription regulator
Cytokine
Kinase
Enzyme
Other
Transmembrane receptor
Upregulated
Downregulated
Direct interaction
Indirect interaction
IL18R1
HERC6
IL12RB2
IL12RB2/IL23R
JAK
IL12 receptor
IL7R
cytokine receptor
CMPK2
IL23R
IFNAR1
IL21
IL23
IL22RA1
TBX21
IgG4
STAT5a/b
Pro-inflammatory Cytokine
CSF2RB
TNFSF15
Stat3-Stat3
IL1RL1
IL22
FIBRINOGEN (family)
SAA
IL-1R
CD3 group
ERK1/2
FCGR3A/FCGR3B
Fcgr3
Cr3
Vla-4
PKC alpha/beta
CD6
JAM3
B
Percent survival
Days post-implantation
Lyz2/Myd88 16 Gy
Lyz2/Myd88 αLy6G 16Gy
Tumor volume (mm³)
3/8
5/12
C
Lyz2/Myd88 16 Gy
Lyz2/Myd88 αCSF1R 16Gy
3/8
6/12
D
Lyz2/Myd88 CTL lipos. 16 Gy
Lyz2/Myd88 Clod. lipos. αCSF1R 16 Gy
3/10
4/7

Supplemental Figure S7. Depletion of Macrophages or Granulocytes does not reverse efficacy of RT in *Lyz2-Cre/Myd88$^{fl/fl}$* mice.

(A) IPA network analysis of significantly differentially regulated genes identified using the PanCancer Immune Profiling gene set by NanoString. Genes upregulated in *Lyz2-Cre/Myd88$^{fl/fl}$* mice are depicted in red, while genes upregulated in *Myd88$^{fl/fl}$* mice are depicted in green, with color intensity associated with degree of upgregulation. Chosen network consists of the top scoring network (score = 37). **(B)** Survival curves (left) and individual Panc02-SIY tumor growth curves (right) in control *Lyz2-Cre/Myd88$^{fl/fl}$* mice treated with 16 Gy RT or depleted of granulocytes with αLy6G (n = 8-12 mice per group). **(C)** Survival curves (left) and individual Panc02-SIY tumor growth curves (right) in control *Lyz2-Cre/Myd88$^{fl/fl}$* mice treated with 16 Gy RT or depleted of macrophages with αCSF1R (n = 8-12 mice per group). **(D)** Survival curves (left) and individual Panc02-SIY tumor growth curves (right) in control *Lyz2-Cre/Myd88$^{fl/fl}$* mice treated with 16 Gy RT and control liposomes or depleted of macrophages with αCSF1R and clodronate liposomes (n = 7-10 mice per group). Significance of differentially regulated genes in **(A)** was defined as fold change >1.5 and $p < 0.1$. Numbers in the upper right of individual tumor growth curves in **(B-D)** represent the proportion of mice cured by RT. Significance in **(B-D)** was assessed log-rank test.
